# Relationships between soil and leaf mineral composition are element‐specific, environment‐dependent and geographically structured in the emerging model *Arabidopsis halleri*

**DOI:** 10.1111/nph.14219

**Published:** 2016-10-13

**Authors:** Ricardo J. Stein, Stephan Höreth, J. Romário F. de Melo, Lara Syllwasschy, Gwonjin Lee, Mário L. Garbin, Stephan Clemens, Ute Krämer

**Affiliations:** ^1^ Department of Plant PhysiologyRuhr University BochumUniversitätsstrasse 150 ND3/30D‐44801BochumGermany; ^2^ Department of Plant PhysiologyUniversity of BayreuthUniversitätsstrasse 30D‐95440BayreuthGermany; ^3^ Bayreuth Center of Ecology and Environmental Research (BayCEER)University of BayreuthUniversitätsstrasse 30D‐95440BayreuthGermany; ^4^ Programa de Pós‐Graduação em Ecologia de EcossistemasUniversidade Vila VelhaRua Comissário José Dantas de MeloBoa Vista29102‐770Vila VelhaEspírito SantoBrasil

**Keywords:** adaptation, ecological speciation, edaphic range, elemental defence, extremophile, heavy metal tolerance, intraspecific trait variation, metal hyperaccumulation

## Abstract

- Leaf mineral composition, the leaf ionome, reflects the complex interaction between a plant and its environment including local soil composition, an influential factor that can limit species distribution and plant productivity. Here we addressed within‐species variation in plant–soil interactions and edaphic adaptation using *Arabidopsis halleri*, a well‐suited model species as a facultative metallophyte and metal hyperaccumulator.
- We conducted multi‐element analysis of 1972 paired leaf and soil samples from 165 European populations of *A. halleri*, at individual resolution to accommodate soil heterogeneity. Results were further confirmed under standardized conditions upon cultivation of 105 field‐collected genotypes on an artificially metal‐contaminated soil in growth chamber experiments.
- Soil‐independent between‐ and within‐population variation set apart leaf accumulation of zinc, cadmium and lead from all other nutrient and nonessential elements, concurring with differential hypothesized ecological roles in either biotic interaction or nutrition. For these metals, soil–leaf relationships were element‐specific, differed between metalliferous and nonmetalliferous soils and were geographically structured both in the field and under standardized growth conditions, implicating complex scenarios of recent ecological adaptation.
- Our study provides an example and a reference for future related work and will serve as a basis for the molecular–genetic dissection and ecological analysis of the observed phenotypic variation.

## Introduction

In addition to carbon dioxide and water for primary production through photosynthesis, land plants require a number of inorganic mineral nutrients that they must take up from the soil solution into roots and partition appropriately within the plant. Edaphic imbalances, which are nutrient deficiencies or the harmful excess of mineral ions in the soil, constrain species composition and the productivity of plant communities (Kruckeberg, 1986; Vitousek *et al*., 2010; Condit *et al*., 2013). Agricultural yield losses attributed to soil nutrient deficiencies in nitrogen, phosphorus, potassium and zinc (Zn), for example, were estimated to exceed 27% worldwide (Tan *et al*., 2005; Alloway, 2008), and the global dependence of industrial agriculture on mineral fertilizers contributes substantially to the depletion of nonrenewable resources (Gilbert, 2009). Hence, a better understanding of how to augment the concentrations of essential nutrients in aboveground plant tissues holds promise for future sustainable crop production and bio‐fortification towards alleviating global malnutrition (http://www.harvestzinc.org/harvestplus).

The mineral composition of plants, also termed the ionome (Salt *et al*., 2008), is an integrated outcome of interactions between endogenous plant processes and the environment (Baxter & Dilkes, 2012). Species‐wide analyses of the relationships between soil composition and the leaf ionome have potential towards understanding edaphic interactions in ecology, as well as identifying physiological strategies and genetic alleles affording local adaptation. However, our present knowledge of these complex plant–environment interactions remains sketchy and limited in particular by the vast and discontinuous variation in soil composition at multiple scales in nature, which renders the spatial resolution of publicly available soil data insufficient (Anderson *et al*., 2016; Pease *et al*., 2016). Joint collections of both plant accessions and adjacent soil were suggested to overcome this bottleneck (Baxter & Dilkes, 2012). As a particularly well‐suited model species towards a general understanding of how the leaf ionome integrates diverse ecological plant–environment interactions, we chose *Arabidopsis halleri*, a wild, perennial, outcrossing and genetically diverse extremophyte in the sister clade of the classical genetic model plant *Arabidopsis thaliana*.

*Arabidopsis halleri*, re‐named from the earlier *Cardaminopsis halleri* (O'Kane & Al‐Shehbaz, 1997), is characteristic among the rare natural colonizers of so‐called metalliferous soils containing high concentrations of heavy metals, such as Zn, cadmium (Cd) and lead (Pb) (Ernst, 1974). In contrast to many edaphic specialists found on metalliferous soils, populations of *A. halleri* are also known on normal, nonmetalliferous soils (Bert *et al*., 2002). Most metalliferous soils have arisen through anthropogenic pollution, which began a few thousand years ago (Brännvall *et al*., 1999) and has accelerated rapidly over the past 150 yr (Nriagu & Pacyna, 1988). Consequently, the recent evolution of heavy metal tolerance in plants may be of general relevance for other instances of rapid or drastic environmental change, such as global warming (Antonovics *et al*., 1971; Schoener, 2011). In addition to metal hypertolerance, *A. halleri* is known for a second, distinct type of a naturally selected extreme trait, metal hyperaccumulation. By definition, concentrations above 3000 μg g^−1^ Zn or above 100 μg g^−1^ Cd were detected in dry leaf biomass of at least one *A. halleri* individual grown in its natural habitat, thus exceeding concentrations in ordinary plants by more than an order of magnitude (Baker & Brooks, 1989; Dahmani‐Muller *et al*., 2000; Bert *et al*., 2002; Krämer, 2010; van der Ent *et al*., 2013). Zn hyperaccumulation can be seen as an exaggerated accumulation of a micronutrient required by all vascular plants at minimal concentrations of *c*. 20 μg g^−1^ leaf dry biomass. By contrast, there is no known nutritional requirement for Cd (Marschner, 1995). Metal and metalloid hyperaccumulation, reported in > 500 species of vascular plants to date, were proposed to act as a defence against biotic stress (Boyd, 2007; van der Ent *et al*., 2013), and this is presently the best‐supported hypothesis also in *A. halleri* (Boyd & Martens, 1992; Kazemi‐Dinan *et al*., 2014, 2015). Elemental defence‐related strategies were also reported in mammals (Festa & Thiele, 2012), suggesting that they may be widespread in biology but usually employed in a highly localized manner and thus undetectable at the bulk tissue level. There is considerable interest in metal hyperaccumulation in basic and applied research towards the development of technologies for phytomining and the re‐cultivation (phytostabilization) or clean‐up (phytoremediation) of heavy metal‐polluted soils (Chaney *et al*., 1997; Salt *et al*., 1998; Verbruggen *et al*., 2009; Krämer, 2010, 2015). Pb and Cd, for example, rank second and seventh, respectively, in the Agency for Toxic Substances and Disease Registry 2015 Priority List of Hazardous Substances (http://www.atsdr.cdc.gov/SPL/) (Clemens *et al*., 2013; Bernhardt & Gysi, 2015).

Here we report the first large‐scale field survey in any plant species of multi‐element composition in paired leaf and soil samples at individual resolution. In agreement with our hypothesis, we identified element‐specific and environment‐specific, as well as geographically diversified, relationships between soil composition and the leaf ionome, and these were reproduced in field‐collected individuals under standardized growth conditions. Soil–ionome relationships differed between the strongly accumulated metals Zn, Cd and Pb on one side, and all other nutrient and non‐nutrient elements on the other side, concurring with their distinct ecological roles. Furthermore, pronounced differences among soil–plant relationships of Zn, Cd and Pb suggested differing ecological and evolutionary trajectories of their accumulation in *A. halleri*. To accommodate the observed complexity, we propose a modified concept for the classification of metal hyperaccumulation in plants. Our study provides an example and a reference for future analogous studies in other species. In combination with the associated unique edaphically and ionomically indexed germplasm collection, this study sets up a model system for addressing intraspecies variation in physiological traits for ecological, evolutionary, molecular mechanistic and applied research.

## Materials and Methods

### Plant and soil sampling in the field

At each of 165 field sites hosting natural populations of *Arabidopsis halleri* (L.) O'Kane and Al‐Shehbaz in Europe (Supporting Information Fig. S1), and from *c*. 12 *A. halleri* individuals per population, we sampled leaves as well as soil from within a 0.05‐m radius around the plant, thus generating a total of 2006 individual pairs of plant leaf and soil samples for the determination of soil pH and multi‐element analysis (see also Methods S1).

### Processing and analysis of samples

Dried leaf samples were microwave‐digested in 65% (w/w) HNO_3_. For an account of total, extractable and exchangeable fractions of soil composition, each air‐dried soil sample was extracted in 3 : 1 (v/v) 37% (w/w) HCl and 65% (w/w) HNO_3_ in a microwave, 0.1 M HCl at room temperature (RT) and 0.01 M BaCl_2_ at RT, respectively. After filtering of soil extracts and acidification using HNO_3_ where necessary, element concentrations (Al, aluminium; B, boron; Ca, calcium; Cd, cadmium; Cr, chromium; Cu, copper; Fe, iron; K, potassium; Mg, magnesium; Mn, manganese; Ni, nickel; P, phosphorus; Pb, lead; S, sulfur; and Zn, zinc) in each extract were determined in triplicate by Inductively Coupled Plasma Atomic Emission Spectrometry (ICP‐AES; iCAP 6500 Duo; ThermoFisher, Dreieich, Germany) employing appropriate quality controls (see Methods S1). For the determination of soil pH, 3 g soil was mixed with 7.5 ml of 0.01 M CaCl_2_ in a 15‐ml round‐bottom polypropylene screw‐cap tube using an overhead shaker (150 rpm at RT) overnight (Hendershot *et al*., 2007). Samples were centrifuged (2000 ***g*** at RT) and pH was determined in the supernatant using a pH meter (WTW pH 522, Wissenschaftlich‐Technische Werkstätten GmbH, Weilheim, Germany).

### Data validation and analysis

After removing soil‐contaminated leaf samples based on a logistic regression model (Baxter *et al*., 2008), we retained leaf–soil sample pairs from 1972 individuals for further analysis (see Methods S1). We used hierarchical (Ward) clustering to classify collection sites into 46 metalliferous and 119 nonmetalliferous sites according to soil HCl‐extractable Zn, Pb, Cu and Cd concentrations that allowed the best supported clustering (Fig. S2, Notes S1; 506 metalliferous and 1466 nonmetalliferous individual soil samples, see Notes S2; see Methods S1). An influence of the timing of sampling on leaf mineral composition was excluded through Principal Component Analyses (Notes S3) and linear regression analyses (see Methods S1). Data analysis and the generation of figures were conducted using the stats v.2.15.3, vegan v.2.0‐10 and plotrix v.3.5‐12 R packages (Peres‐Neto *et al*., 2006; R Core Team, 2013; see Methods S1).

### Zn and Cd accumulation phenotyping in the growth chamber

Seven or more months after transfer to our growth facilities, five replicate cuttings were generated from each of 105 mother plants, and clones were allowed to grow roots for 15 d. Plants were transplanted into pots containing *c*. 300 g of an experimental soil mix consisting of 2 : 1 loamy soil:sand mix amended with 300 mg Zn kg^−1^ (added as ZnS) and 5 mg Cd kg^−1^ (added as CdCl_2_ × H_2_O) and cultivated in a climate‐controlled growth chamber. Pots were watered as needed onto the soil surface (*c*. 10 ml ultrapure water every 5 d) and re‐randomized in 2‐weekly intervals. After 6 wk on experimental soils, entire shoots were harvested, washed in ultrapure water, dried, homogenized, and subsamples were digested for multi‐element analysis as described above (see Methods S1).

## Results

We conducted a field survey at individual resolution of both leaf and soil elemental composition in *Arabidopsis halleri*, covering 46 metalliferous and 119 nonmetalliferous sites from sea level to 2250 m above sea level (asl) and all four European subspecies *halleri*,* ovirensis*,* tatrica* and *dacica* (Koch & Matschinger, 2007) (Figs S1a, S2; Notes S1). From each collection site, approximately half of the sampled individuals were transferred to our greenhouse for vegetative propagation in order to establish an edaphically and ionomically indexed biodiversity collection of this stoloniferous and self‐incompatible perennial species. According to soil composition and soil pH, the edaphic range of *A. halleri* appeared surprisingly broad overall, as well as among metalliferous or nonmetalliferous soils alone (Fig. S1b–m; Notes S2). Within sites, soil composition was highly patchy (see Notes S2). Far more populations of *A. halleri* than expected were found on nonmetalliferous soils (see Fig. S2). Note that previous studies classified sites as metalliferous or nonmetalliferous based on the vegetation type determined according to indicator plant species, or based on the total concentration of a single metal in the soil exceeding a threshold (Bert *et al*., 2002). For this study, we employed a quantitative, hierarchical clustering approach including BaCl_2_‐exchangeable concentrations of Zn, Cd, Pb and Cu for an operational classification of sites (Methods S1; Fig. S2). Depending on the research question addressed, a specifically adapted classification approach may be useful.

**Figure 1:**
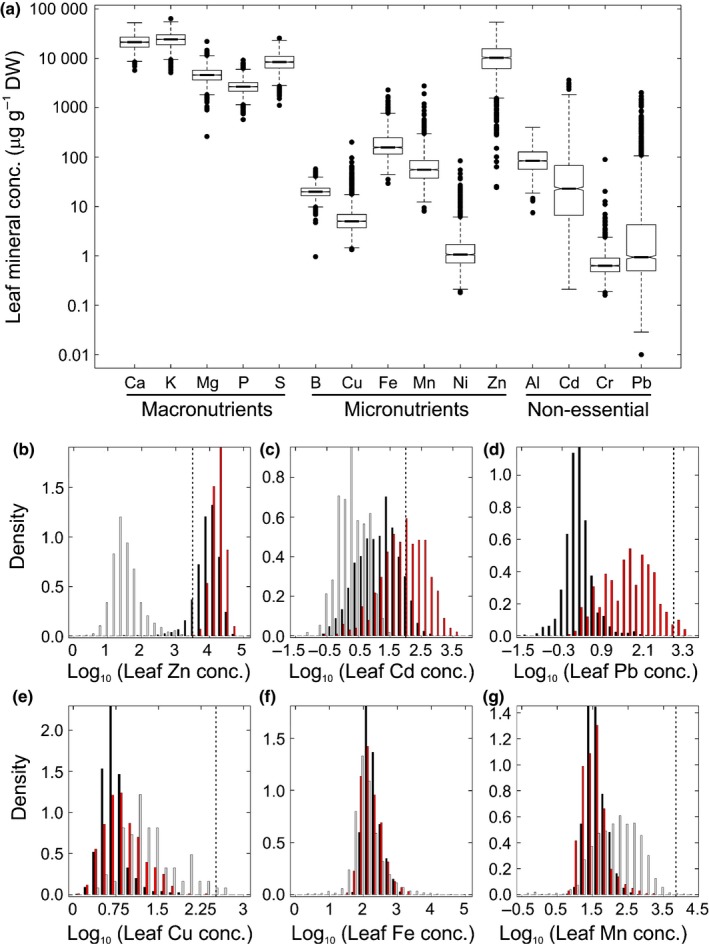
Element concentrations in leaves of *Arabidopsis halleri* sampled at their natural sites of growth. (a) Leaf concentrations of macronutrients, micronutrients and nonessential elements. Al, aluminium; B, boron; Ca, calcium; Cd, cadmium; Cr, chromium; Cu, copper; Fe, iron; K, potassium; Mg, magnesium; Mn, manganese; Ni, nickel; P, phosphorus; Pb, lead; S, sulfur; and Zn, zinc. Shown is the median (central horizontal line) with 25/75 percentiles (boxes), 10/90 percentiles (bars), and outliers (closed circles; > 1.5‐fold the interquartile range above/below the upper/lower quartile) (*n *=* *1972 plant individuals). (b–g) Density histograms of leaf concentrations of Zn, Cd, Pb, Cu, Fe and Mn in *A. halleri*. Log_10_‐transformed data (class width 0.2) are shown for metalliferous (*n *=* *506; red) and nonmetalliferous (*n *=* *1466; black) soils, with a published multi‐species field survey (grey) shown for reference (Watanabe *et al*., 2007). Dotted vertical lines mark thresholds for metal hyperaccumulation (Zn, 3000 μg g^−1^; Cd, 100 μg g^−1^; Pb, 1000 μg g^−1^; Cu, 300 μg g^−1^; Mn, 10 000 μg g^−1^). The published dataset (Watanabe *et al*., 2007) included data for leaf Zn (*n *=* *2193), Cd (*n *=* *246), Cu (*n *=* *19), Fe (*n *=* *2039) and Mn (*n *=* *2182) concentrations.

We observed higher concentrations than reported among land plants so far of up to 53 900 μg Zn g^−1^ dry leaf biomass at a nonmetalliferous site (Kowari/PL) and 3640 μg Cd g^−1^ dry leaf biomass at a metalliferous site (Tarnowskie Góry/PL), clearly stemming from physiological accumulation processes (342 μg g^−1^ Zn and 896 μg g^−1^ Cd in soil total fraction, respectively; Notes S2). Additionally, Pb concentrations reached hyperaccumulator levels above 1000 μg g^−1^ leaf dry biomass at three metal‐contaminated sites (Miasteczko Śląskie/PL, 10 individuals; Steinbachrotte/AT, 3 individuals; Mount Hochobir/AT, 1 individual; Fig. 1a; see Notes S2). Although a series of physiological experiments will be required to verify our preliminary observation of Pb hyperaccumulation in *A. halleri* (Baker, 1981; van der Ent *et al*., 2013), the species is undoubtedly capable of exceptionally high accumulation of Pb in leaves.

### Intraspecies variation in leaf mineral element concentrations contrasts between ordinary minerals and strongly accumulated metals, and also among the latter

Leaf concentrations of the metals strongly accumulated by *A. halleri*, namely Pb, Cd and Zn, spanned 5.3, 4.2 and 3.4 orders of magnitude (Fig. 1a; Notes S2), respectively, a considerably larger range than for all other elements quantified in this study (macronutrients 1.0 to 1.9, micronutrients and others 1.8 to 2.7 orders of magnitude, respectively). Major differences were also observed among intraspecies variation in Zn, Cd and Pb accumulation (Fig. 1b–d). The frequency distribution of concentrations of the micronutrient Zn in leaves of *A. halleri* showed a maximum > 300‐fold above leaf Zn concentrations in other vascular plant species (Watanabe *et al*., 2007), spanning a concentration range that was almost entirely distinct and generally characteristic of macronutrients such as Ca, K and S (Fig. 1a,b). This resembles classical multi‐species surveys of hyperaccumulator and nonhyperaccumulator species with a single or a few datapoints per species (Brooks, 1987). Hyperaccumulator concentrations above 3000 μg Zn g^−1^ dry leaf biomass (Broadley *et al*., 2007; Krämer, 2010) were detected in one or more individuals from each sampled population (Notes S2), which is concordant with constitutive (species‐wide) hyperaccumulation of Zn in *A. halleri* (Bert *et al*., 2000) in the much larger set of populations analysed here from a broader geographical range. Leaf Zn concentrations were below hyperaccumulator levels in only 8% of individuals, whereby Zn concentrations were as low as the nonaccumulator range only for a very small subset of individuals exclusively from two populations (see Fig. 5b later). For concentrations of the nonessential Cd in leaves of *A. halleri*, the frequency distribution was broadened substantially in comparison to that of leaf Zn concentrations. Its maximum was only at *c*. 30‐fold higher Cd concentrations than that of the frequency distribution in the multi‐species survey of vascular plants used for comparison (Watanabe *et al*., 2007). Leaf Cd concentrations of *A. halleri* ranged from concentrations that are common among vascular plants up to exceptionally high concentrations more than two orders of magnitude above those and clearly above typical micronutrient concentrations in leaves (Fig. 1a,c). This differed from an expected classical frequency distribution associated with hyperaccumulation (Brooks, 1987) (see earlier, Fig. 1b). Hyperaccumulator concentrations of > 100 μg g^−1^ Cd were detected in 18.3% of all individuals and in at least one individual in a total of 70 populations out of the 165 populations sampled in this study (see also Notes S2). Hyperaccumulation of Cd is thus not a species‐wide trait in *A. halleri*, if – as for Zn above – we apply the definition of hyperaccumulation at the population level, rather than the species level at which metal hyperaccumulation was defined (Baker & Brooks, 1989). Remarkably, we detected leaf Cd hyperaccumulation in 8.1% of *A. halleri* individuals collected on nonmetalliferous soils that contain only trace amounts of Cd (see also Notes S2). This suggested an extremely high efficacy of Cd enrichment in the leaves of these individuals, despite the fact that Cd – in contrast to Zn – does not have a nutritional role in any land plant. Different from Zn and Cd, the frequency distribution of leaf Pb concentrations in *A. halleri* was bimodal, with distinct peaks on metalliferous and nonmetalliferous soils. On metalliferous soils the most frequently detected Pb concentration was more than 30‐fold higher than on nonmetalliferous soils (Fig. 1d). Hyperaccumulator concentrations of > 1000 mg g^−1^ Pb were detected in leaves of 0.7% of sampled individuals, all of which originated from metalliferous soils (Notes S2; see earlier). Contrary to Zn, Cd and Pb, the concentrations of the micronutrients Cu and Mn were at the lower end of, and for Fe within, the concentration ranges established for field‐collected leaf tissues from a variety of other vascular plant species (Fig. 1e–g).

Median leaf concentrations at metalliferous sites were higher for Zn, Cd, Pb and Cu, and lower for macronutrients P, K and S, than at nonmetalliferous sites (Fig. S3). Across our sampling range, *A. halleri* individuals thus accommodated highly diverse internal metal concentrations, and in metalliferous‐type habitats a combination of several internal physiological challenges in addition to high external heavy metal concentrations in the soil solution. These observations warrant a differentiated, element‐ and habitat‐specific concept of metal hyperaccumulation and associated hypertolerance. A Principal Component Analysis of mineral concentrations in leaves identified groups of co‐varying elements, whereby an evident influence of soil type warranted the analysis of interelement relationships in the context of soil mineral composition (Fig. S4; Notes S4).

### Soil composition explains intraspecies variation in leaf composition only partially and to differing extents depending on the element

**Figure 2:**
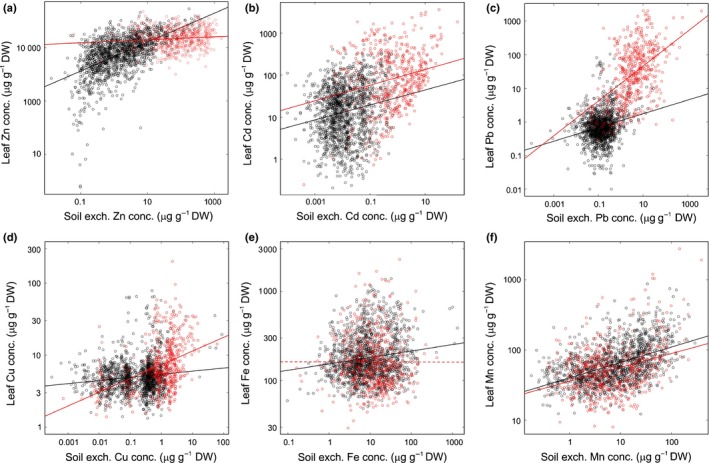
Relationships between element concentrations in leaves and local soil of *Arabidopsis halleri* at its natural sites of growth. (a) Zinc (Zn), (b) cadmium (Cd), (c) lead (Pb), (d) copper (Cu), (e) iron (Fe), and (f) manganese (Mn). Each datapoint represents one *A. halleri* individual (red: metalliferous soils, *n *=* *506; black: nonmetalliferous soils, *n *=* *1466), with leaves and adjacent soil (BaCl_2_‐exchangeable concentrations). Linear regression models are given for each soil type (continuous lines *P *<* *0.05; dotted trendline not significant); see Supporting Information Table S2).

Redundancy analyses (RDA) suggested that, globally, variation in soil exchangeable concentrations accounted for a larger proportion of variation in leaf element concentrations (adjusted *R* ^*2*^ = 0.271; *P *<* *0.005), than total or HCl‐extractable soil fractions (Table S1). By comparison to the vast range of variation in soil composition, leaf concentrations were generally maintained within a narrower range (Fig. 2). We detected positive linear correlations of leaf concentrations with soil exchangeable concentrations for Zn, Cd and Pb, and also for the nutrients Cu, Fe and Mn, with Fe on metalliferous soils as the only exception (Table S2). For Zn, however, leaf concentrations increased only little across a wide range of soil exchangeable Zn concentrations, with a strong dependence on soil exchangeable Zn concentrations exclusively at the lowest end of the concentration range (Fig. 2a). By contrast, we observed a pronounced linear increase in leaf Pb concentration with increasing soil exchangeable Pb concentrations for *A. halleri* individuals sampled on metalliferous soils (Fig. 2c). Leaf Cd concentrations showed an intermediate dependence on the exchangeable fraction of Cd in the soil, with a remarkably broad range of leaf Cd concentrations in the presence of low soil Cd concentrations (Fig. 2b). This indicated that there were other factors influencing leaf mineral composition, most notably in Cd and Zn. A comprehensive assessment of the complex multi‐factorial relationships between soil and plant composition by RDA supported the conclusion that soil mineral composition explains only part of the variation in leaf Zn and Cd concentrations (Fig. S5; Notes S4; Table S3). Moreover, several known relationships between soil and leaf composition were identified, corroborating our analysis.

**Figure 3:**
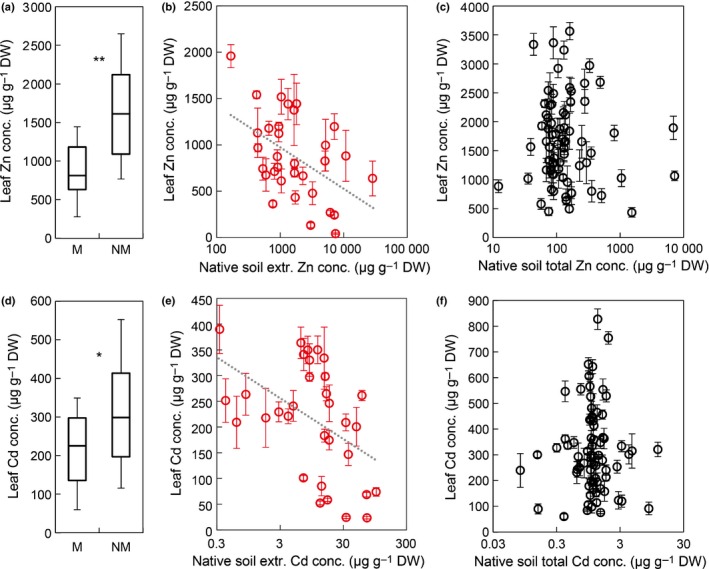
Leaf zinc (Zn) and cadmium (Cd) concentrations in individuals of *Arabidopsis halleri* cultivated under standardized controlled growth chamber conditions. (a) Median leaf Zn concentrations (25/75 percentiles, bars 10/90 percentiles) of individuals originating from metalliferous (M, *n *=* *32 individuals) and nonmetalliferous soils (NM,* n *=* *73 individuals). (b) Leaf Zn concentration for each individual in relation to local HCl‐extractable soil Zn concentration at its site of origin in the field (mean ± SD,* n *=* *5 vegetative clones per individual) for M soils as summarized in (a). (c) Leaf Zn concentration for each individual in relation to local total soil Zn concentration at its site of origin in the field (mean ± SD,* n *=* *5 vegetative clones) for NM soils as summarized in (a). (d) Median leaf Cd concentrations (25/75 percentiles, boxes; 10/90 percentiles bars) of individuals originating from metalliferous (M, *n *=* *32 individuals) and nonmetalliferous soils (NM,* n *=* *73 individuals). (e) Leaf Cd concentration for each individual in relation to local HCl‐extractable soil Cd concentration at its collection site in the field (mean ± SD,* n *=* *5 vegetative clones per individual) for M soils as summarized in (d). (f) Leaf Zn concentration for each individual in relation to local total soil Zn concentration at its collection site in the field (mean ± SD,* n *=* *5 vegetative clones) for NM soils as summarized in (d). *Arabidopsis halleri* individuals were propagated vegetatively, transplanted into a soil amended with Zn and Cd at nontoxic concentrations, and harvested after 6 wk. Red and black symbols denote individuals from M and NM soil origin, respectively (b, c, e, f). Dotted trendlines are *y *=* *−191 log_e_(*x*) + 2290, *R* ^2^ = 0.23, *P *<* *0.01 (b); *y *=* *34.1 log_e_(*x*) + 292, *R* ^2^ = 0.22, *P *<* *0.01 (e). *, *P *<* *0.01; **, *P *< 10^−6^ (Wilcoxon rank sum test with Bonferroni corrections for multiple comparisons).

We hypothesized that differences between plant individuals may contribute to the proportion of variation in leaf metal accumulation that was unaccounted for by variation in soil composition. To examine this experimentally, leaf metal accumulation was quantified in vegetative clones of a subset of 105 individuals collected in the field upon cultivation in a metal‐amended nontoxic experimental soil under controlled growth conditions (Table S4). This confirmed that genotypes originating from metalliferous soils accumulated lower concentrations of Zn and Cd in leaves than individuals originating from nonmetalliferous soils, with 50% and 24% lower medians for Zn and Cd, respectively (Fig. 3a,d; Notes S5), consistent with our observations in the field (see Fig. 2) and a published small‐scale study (Bert *et al*., 2002). Among plant individuals collected at metalliferous sites, there were overall negative relationships between leaf metal accumulation in the growth chamber experiment and metal concentrations in the soil of origin of the respective plant individual (Fig. 3b,e). Consequently, the higher the metal concentration in the metalliferous soil of origin of an individual, the lower was its metal accumulation in our experiment. Notwithstanding this overall relationship, there were individuals of strongly differing metal accumulation properties. However, among individuals originating from nonmetalliferous soils, leaf Zn and Cd accumulation were even more highly differentiated in our experiment. This was observed, in particular, among individuals originating from soils containing intermediate concentrations of metals of between 12 and 170 mg kg^−1^ Zn and 0.08 and 2 mg kg^−1^ Cd in the soil total fraction (Fig. 3c,f). Growth chamber data were independently reproducible (Fig. S6a,b). These results confirmed that metal accumulation properties observed experimentally in plant genotypes are related to metal concentrations in their soils of origin, in a manner differing fundamentally between metalliferous and nonmetalliferous soil types.

### Biogeographical signatures of variation in trait values

**Figure 4:**
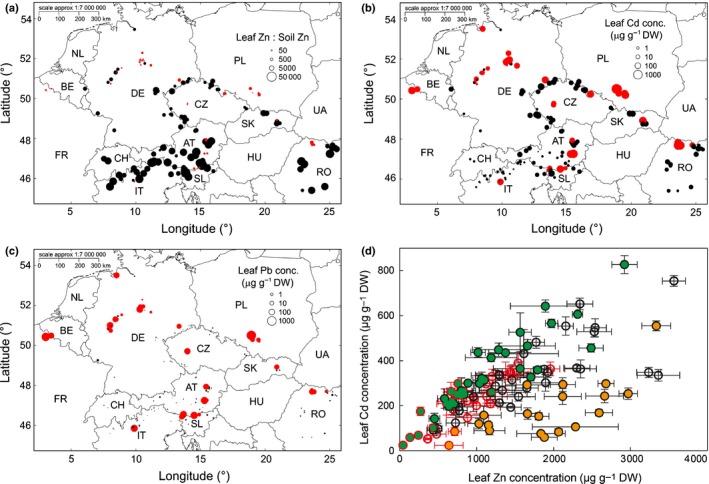
Biogeographical signatures of metal accumulation. (a–c) Maps showing population median of leaf zinc (Zn) accumulation efficiency (ratio of leaf Zn to soil exchangeable Zn concentration, a), leaf cadmium (Cd) concentration (b), and leaf lead (Pb) concentration (c) in *Arabidopsis halleri* sampled at natural sites of growth in the field. Note that ratios become inaccurate where the concentration of an element in soil approaches zero. (d) Relationship between leaf Cd and Zn concentrations of *A. halleri* cultivated under standardized controlled growth chamber conditions. Red symbols/borders, metalliferous sites/soil origin; black symbols/borders, nonmetalliferous site/soil origin (a–d). Each datapoint (d) represents one individual (mean ± SD,* n *=* *5 vegetative clones per individual; see also Fig. 3). Orange circles, Zn‐to‐Cd ratio > 6.91 (75^th^ percentile) and population originating from the South (IT, Italy; CH, Switzerland; SL, Slovenia; AU, Austria compare a). Green circles, Zn‐to‐Cd ratio < 3.53 (25^th^ percentile) and population originating from the East or North (DE, Germany; CZ, Czech Republic; SK, Slovakia; PL, Poland; RO, Romania; FR, France; BE, Belgium, compare b). Percentiles refer to all individuals from nonmetalliferous soil origin, and a uniform colour was applied to all individuals of a population when at least one individual met the quantitative criteria (see Supporting Information Notes S5).

In order to further examine soil‐independent variation in metal accumulation, we addressed whether leaf metal accumulation in the field exhibited any spatial structure at a geographical scale. The highest ‘Zn accumulation efficiencies’, calculated as the population median of the ratios of leaf Zn to soil exchangeable Zn concentration, of up to 38 900 (Lozio/IT), were geographically enriched on nonmetalliferous sites south of the Alpine divide and in Romania, compared to a minimum of 339 (Zakopane, Zapa/PL) on nonmetalliferous sites in the North (Fig. 4a). For Cd accumulation, we observed a distinct geographic pattern. Among nonmetalliferous sites, the highest leaf Cd concentrations were geographically enriched in the mountain ranges along the western and northern borders of the Czech Republic with Germany and southeastwards into Romania (Fig. 4b). Here, population medians of up to 170 μg g^−1^ dry biomass (Thermalbad Wiesenbad/DE) contrasted strongly with low leaf Cd accumulation south of the Alpine divide (see Fig. 4b) down to a minimum of 0.74 μg g^−1^ dry biomass (Intragna/CH). High leaf Pb concentrations were detected at some scattered sites characterized by high concentrations of anthropogenic soil contamination (e.g. Évin Malmaison/FR, Rabenstein Castle/AT; Fig. 4c). Leaf Pb accumulation was low at other metalliferous sites exhibiting similar or even higher concentrations of soil Pb contamination (Haufenreith/AT, Băile Borşa/RO). Thus, high concentrations of leaf Pb accumulation were restricted to a geographically dispersed subset of Pb‐contaminated sites.

Next we examined whether the biogeographical patterns in leaf Zn and Cd accumulation apparent from our field data were experimentally reproducible under controlled growth conditions. Of the individuals constituting the quartile of the highest leaf Zn relative to Cd accumulation in the growth chamber experiment, 82% originated from south of the Alpine divide (Fig. 4d; Zn : Cd ratio > 6.91). Conversely, of the lower quartile of individuals accumulating the lowest Zn relative to Cd concentrations in our experiment, all originated from the north and east of our sampling range (Zn : Cd ratio < 3.53). We concluded from this that it was possible to identify novel reproducible biogeographical patterns of within‐species variation in leaf metal accumulation based on our analysis of field‐collected leaf and soil samples (see also Fig. S6a,b).

### Within‐population variation in trait values

**Figure 5:**
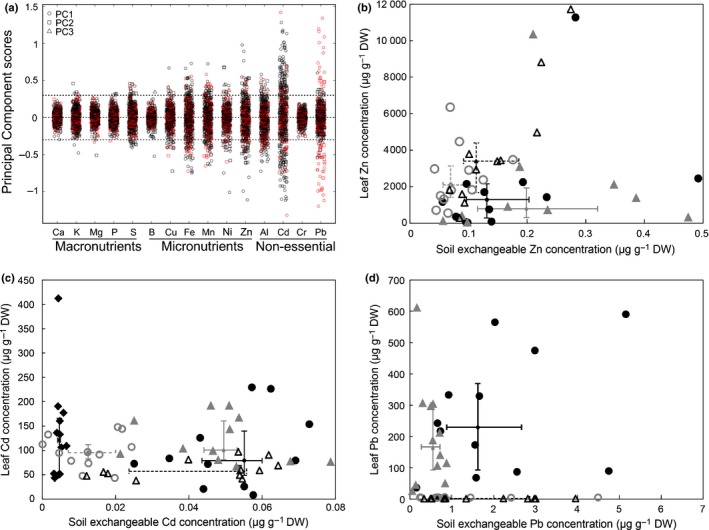
Variation in leaf element concentrations within natural populations of *Arabidopsis halleri* sampled in the field. (a) Overview of within‐population variation in leaf element concentrations. Al, aluminium; B, boron; Ca, calcium; Cd, cadmium; Cr, chromium; Cu, copper; Fe, iron; K, potassium; Mg, magnesium; Mn, manganese; Ni, nickel; P, phosphorus; Pb, lead; S, sulfur; and Zn, zinc. Each datapoint reflects the magnitude of residual within‐population variation in leaf element concentration that is unexplained by variation in soil composition, for each population sampled at a metalliferous (red) or nonmetalliferous (black) site. (b–d) Examples of leaf metal concentrations shown in relation to local soil exchangeable metal concentrations for the individuals of populations of contrasting variation in Zn (b), Cd (c) and Pb (d) accumulation. Populations Idrija/SL (Slovenia) (closed circles, *n *=* *12) and Hartelsberg/AT (Austria) (closed triangles, *n *=* *10), and populations Poppermig/AT (open circles, *n *=* *12) and Sankt Leonhard/IT (Italy) (open triangles, *n *=* *11); all nonmetalliferous (b). Populations Ukanc/SL (closed diamonds, *n *=* *12), Bad Gottleuba/DE (Germany) (closed circles, *n *=* *11) and Runding/DE (closed triangles, *n *=* *12), Zakopane/PL (Poland) (open circles, *n *=* *12) and Regenstauf/DE (open triangles, *n *=* *12), all nonmetalliferous (c). Populations Rabenstein Castle/AT (closed circles, *n *=* *12) and Gailitz/AT (closed circles, *n *=* *12), Lgota/PL (open circles, *n *=* *12) and Eckertal/DE (open triangles, *n *=* *12), all metalliferous (d). Smaller‐size symbols and bars show population medians and 25/75 percentiles (solid, closed‐symbol series; dotted, open‐symbol series), respectively (b–d). For (a), we first developed a global redundancy analysis (RDA) model across all samples at all sites, employing the standardized log_10_(*x *+* *1) leaf element concentrations as a function of soil composition, that is, the standardized log_10_(*x *+* *1) soil exchangeable element concentrations and standardized soil pH (*n *=* *1972; see Supporting Information Fig. S5; standardization through *z*‐scores). Subsequently, we used the residuals from the RDA model to conduct a principal component analysis (PCA) for each of the 165 collection sites, and the resulting principal component scores are shown (PC1, circles; PC2, squares; PC3, triangles). In (b), two outlier datapoints are not shown: Sank_11 (*x *=* *6.66; *y *=* *28 400) and Hart_7 (*x *=* *6.19; *y *=* *313).

All populations maintained soil‐independent residual variation in the leaf ionome within a narrow range for macronutrient, micronutrient and almost all of the nonessential trace elements, most stringently for Cr, B, Ca, Mg, P and K (Fig. 5a). The vast majority of populations also maintained low variation for the micronutrient heavy metal Zn that is hyperaccumulated in *A. halleri* species‐wide, with only few exceptions. By comparison, different subsets of populations exhibited high degrees of within‐population variation in leaf Cd and Pb concentrations, respectively. Enhanced within‐population variation in leaf Cd concentrations was observed predominantly in some populations at nonmetalliferous sites. By contrast, populations exhibiting enhanced variation in leaf Pb concentrations were located at metalliferous sites. These observations further underline fundamental differences in intraspecies variation between hyperaccumulated metals and other elements in *A. halleri* (see also Figs 1, 2).

A few representative examples (Fig. 5b–d) illustrate some of the contrasts summarized in Fig. 5(a). In two populations on nonmetalliferous soils, Idrija/SL and Hartelsberg/AT, several individuals contained surprisingly low, nonhyperaccumulator Zn concentrations of between 24 and 300 μg g^−1^ Zn, whereas other individuals contained hyperaccumulator concentrations of Zn. By contrast, on other soils of comparable exchangeable Zn content, leaf Zn accumulation was generally higher and uniform (Fig. 5b). Among highly Cd‐hyperaccumulating populations on nonmetalliferous soils (Fig. 5c), within‐population variation in leaf Cd concentrations was generally large in the populations Ukanc/SL, Bad Gottleuba/DE and Runding/DE, and contrasting with lower variation in the populations Zakopane (Zako)/PL and Regenstauf/DE. On metalliferous soils, leaf Pb concentrations were very low in all individuals of the populations Lgota/PL and Eckertal/DE, whereas leaf Pb accumulation was high and more variable among individuals in the populations Gailitz/AT and Rabenstein Castle/AT (Fig. 5d).

## Discussion

We observed an exceptionally broad edaphic range for *Arabidopsis halleri*, suggesting multiple and diverse instances of local adaptation (Figs S1b‐m, S2) and pinpointing contrasted populations for future targeted experimental studies (Notes S2). A comparative evaluation of the edaphic range of *A. halleri* will only become possible once similar datasets are available for other plant species (van der Ent *et al*., 2016). Soil composition is thought to be patchy (Kruckeberg, 1986; Baxter & Dilkes, 2012; Condit *et al*., 2013), and our results stress this empirically, thus supporting the exemplary sampling design employed here, rather than the use of existing data available only at coarse resolution (Anderson *et al*., 2016; Pease *et al*., 2016).

The majority of *A. halleri* populations were located on nonmetalliferous soils, which is uncommon among the metal hyperaccumulator species examined at sufficient depth to date (van der Ent *et al*., 2013; Pollard *et al*., 2014). Not only was zinc (Zn) hyperaccumulation ubiquitous in *A. halleri* at nonmetalliferous sites despite only ordinary soil Zn concentrations, but a geographically confined set of *A. halleri* populations was even capable of hyperaccumulating cadmium (Cd) from only very low concentrations in the soil (Fig. 1). Species‐wide relationships between mineral concentrations in soil and leaves differed pronouncedly among the strongly accumulated metals Zn, Cd and lead (Pb), and they also differed strongly between this group of strongly accumulated metals and other nutrient and non‐nutrient minerals (Fig. 2). At natural sites of growth of *A. halleri* in the field, internal concentrations of heavy metals Zn, Cd and Pb in leaves reached concentrations that were far above nutritional requirements attributable to the metal cofactor needs of all internal metalloproteins (see Fig. 1). In fact, there is no known requirement for Cd or Pb in land plants (Marschner, 1995). These are extreme traits by which *A. halleri* differs fundamentally from the species targeted by previous large‐scale studies of multi‐element composition, including the genetic model plant *A. thaliana*, which were conducted in standardized environments (Baxter *et al*., 2008, 2012; Baxter, 2009; Atwell *et al*., 2010; Conn *et al*., 2012). Compared to nonmetalliferous sites, *A. halleri* populations at metalliferous sites generally exhibited lower Zn accumulation efficiencies (see Figs 2, 4) (Bert *et al*., 2000, 2002). This was also visible, although less pronounced, for leaf Cd accumulation, and both findings were confirmed under standardized growth conditions employing *A. halleri* individuals collected in the field (Fig. 3). If we interpret these observations as an outcome of selection, then the selection acting on Zn, Cd and Pb in the leaf ionome of *A. halleri* is complex in comparison to other mineral elements and also in relation to what is known in nonhyperaccumulator species so far (Baxter, 2009; Baxter *et al*., 2012; Chao *et al*., 2012; Lowry *et al*., 2012; Poormohammad Kiani *et al*., 2012).

Given the scope of this study, our findings warrant an adjustment of previously employed concepts. We propose the future use of the terms obligate *vs*. facultative metallophyte for edaphic requirements also of hyperaccumulator species (Pollard *et al*., 2014). Furthermore, we propose to employ the term ‘facultative hyperaccumulation’ exclusively for plant species that comprise both hyperaccumulating and nonhyperaccumulating populations for a given metal (van der Ent *et al*., 2013). Independently of this, it appears useful to distinguish between ‘opportunistic hyperaccumulation’ on metalliferous soils that contain highly elevated concentrations, and ‘extremogenic hyperaccumulation’ on nonmetalliferous soils that contain only background concentrations of the hyperaccumulated metal. Accordingly, *A. halleri* is a hyperaccumulator of Zn and a facultative hyperaccumulator of Cd and possibly Pb, with both opportunistic and extremogenic hyperaccumulation of Zn and Cd, as well as opportunistic accumulation of Pb.

Abundance, frequency and geographical distribution of metal hyperaccumulation in *A. halleri* can be taken to suggest that Zn hyperaccumulation evolved earliest at the base of the *A. halleri* lineage (Figs 1, 2, 4), whereas Cd hyperaccumulation evolved more recently, remains regionally confined and is notably missing in most populations south of the Alpine divide. Interestingly, this southern region corresponds to a phylogeographically distinct unit (Pauwels *et al*., 2012) which we also found to be part of a larger zone of the highest Zn accumulation efficiencies that additionally includes the southeastern phylogeographic unit as well as a hybrid zone between this and a vicariant northwestern phylogeographic unit (Pauwels *et al*., 2012) (Fig. 4). The differential, macro‐geographically structured Zn and Cd accumulation properties evident in *A. halleri* on nonmetalliferous soils were reproducible under standardized growth conditions, and they overlap only partially with the established phylogeographical structure, thus implying recent adaptive evolution (Pauwels *et al*., 2012). Because of the geographically scattered and recent anthropogenic origin of most metalliferous soils, *A. halleri* is thought to have colonized metalliferous sites from nearby populations at nonmetalliferous sites, a hypothesis supported by a regional‐scale population genetic study (Pauwels *et al*., 2005). Our results are consistent with this, and they further suggest that there are diverse physiological strategies of enhanced metal hypertolerance in populations at metalliferous sites. These strategies include the dampening of leaf Zn accumulation observed generally to differing degrees, and the divergent dampening or alternatively internal tolerance of very high leaf Cd and Pb accumulation (Figs 3, 5a).

Especially the biological role of Zn and Cd hyperaccumulation in *A. halleri* on nonmetalliferous soils remains an intriguing question. It is conceivable that local natural selection may act directly to favour heightened heavy metal concentrations in leaves of *A. halleri* (see Fig. 3c,f), for example towards elemental defence against biotic stress that is gaining increasing support by experimental studies (Boyd, 2007; Fones & Preston, 2013; Kazemi‐Dinan *et al*., 2014). Although generally effective (Galeas *et al*., 2008; Quinn *et al*., 2010), elemental defence can be overcome, as was demonstrated by an incidence of tolerance in an insect herbivore towards the hyperaccumulation of selenium (Freeman *et al*., 2006). The bio‐toxicity of Cd is far more severe than that of Zn, invoking the possibility of an ‘arms race’ scenario, by which herbivore resistance to Zn hyperaccumulation may have led to the evolution of Cd hyperaccumulation. Alternatively, the hyperaccumulation of both Zn and Cd in combination could be advantageous because of their additive toxic effects on herbivores (Kazemi‐Dinan *et al*., 2014), and it is presently unknown whether different heavy metals might be differentially effective against different herbivores or even pathogens (Fones *et al*., 2010; Fones & Preston, 2013). Herbivore resistances to Zn and Cd hyperaccumulation are more likely to evolve at metalliferous sites, where highly elevated concentrations of these heavy metals are ubiquitous in soil and water. It is thus interesting to note that, although uncommon, high concentrations of leaf accumulation of the severely biotoxic heavy metal Pb occurred at a small subset of metalliferous sites. We speculate that high leaf Pb accumulation in *A. halleri* arose most recently and locally, after the colonization of metalliferous sites. These ideas will require extensive experimental testing in the future.

In order to counteract the evolution of insect resistances against pesticides, agricultural crop protection applies different toxins in combination (Alstad & Andow, 1995; Zhao *et al*., 2003). Alternatively, crop protection implements the refuge strategy, by which stands of a crop containing an endogenously produced or externally applied pesticide are neighbouring refuges of pesticide‐free plants of the same crop (Tabashnik *et al*., 2008; Carriere *et al*., 2012). In this study, we detected co‐hyperaccumulation of Cd alongside Zn in a number of populations (Notes S2). Moreover, subsets of populations exhibited very high degrees of soil‐independent within‐population variation in leaf accumulation of Cd or Pb, whereas variation in leaf concentrations of other elements quantified in this study remained within a narrow range (Fig. 5). The intriguing possibility that naturally evolved toxin combinations (Kazemi‐Dinan *et al*., 2014) or refuge strategies sustain the efficacy of elemental defences in *A. halleri* warrant future examination. Insect herbivory, mechanical leaf wounding and rhizosphere microbes were shown to trigger increased leaf Cd accumulation in *A. halleri* in controlled experiments (Muehe *et al*., 2015; Plaza *et al*., 2015). The moderate magnitudes of these responses reported so far, however, appear insufficient to explain the quantitatively large extent of within‐population variation in Cd accumulation observed here at a number of field sites. The insights obtained in this study allow an informed design of targeted physiological, genetic and field studies to examine this further.

Phenotypic variation within the species *A. halleri* encompasses remarkable abilities for the differential selective enrichment of the three chemically similar heavy metals Zn, Cd and Pb (Fig. 4), which generally share pathways of movement within plants (Krämer, 2009). Overall, there was only a weak positive interrelationship between leaf Zn and Pb concentrations on metalliferous soils (Fig. S5b), and a slightly more pronounced positive relationship between leaf Cd and Pb concentrations on nonmetalliferous soils (Fig. S5c). In the analysis of these field data, an influence of local soil composition on the relationships detected between the accumulation of different elements in leaves can be revealed, but not eliminated. In addition, environmental parameters outside those included in the data analysis, as well as temporal fluctuations in environmental parameters, can have important effects. Consequently, only experiments under standardized growth conditions can provide information on global, physiologically governed interelement relationships across genotypes, and identify outlier genotypes deviating from these (Figs 3, 4d). For example, under standardized conditions we observed here an approximately linear relationship between leaf Zn and Cd concentrations across genotypes, whereby importantly, genotypes of specific regional origins deviated from this overall relationship (Fig. 4d). These and similar analyses in the future will provide a path towards the identification of the genetic basis of the substantial intraspecies phenotypic variation within *A. halleri*. The differential accumulation of Zn, Cd and Pb from soil by *A. halleri* and its variation between genotypes require highly efficient and metal‐specific physiological acquisition, within‐plant partitioning, and physiological tolerance mechanisms, which have potential towards a variety of applications in phytoremediation, phytomining, biofortification and nutritional crop safety. All in all, our field sampling approach allowed a more comprehensive and precise account of intraspecies variation in soil–ionome relationships than has been possible so far. This study, in association with the unique ionomically and edaphically indexed germplasm collection established here, renders *A. halleri* an exquisite model for future studies of ecological speciation, local adaptation, parallel evolution and ecological and evolutionary dynamics.

## Author contributions

U.K. designed the study with S.C. and R.J.S., planned the manuscript with S.C. and R.J.S., wrote the manuscript with contributions from R.J.S. and L.S., edited the manuscript with contributions from all authors; R.J.S. coordinated, planned with S.H., U.K., S.C. and J.R.F.M. and conducted sampling and experiments with S.H. supported by J.R.F.M. and G.L., and analysed data with U.K., M.L.G., S.H., L.S. and S.C.

## Supporting information

Please note: Wiley Blackwell are not responsible for the content or functionality of any Supporting Information supplied by the authors. Any queries (other than missing material) should be directed to the *New Phytologist* Central Office.

**Fig. S1 **Map of European sampling sites and edaphic range of *Arabidopsis halleri*.
**Fig. S2** Classification of sampling sites into metalliferous and nonmetalliferous according to soil composition.
**Fig. S3** Comparison of leaf element concentrations between *Arabidopsis halleri* populations at nonmetalliferous and metalliferous sites.
**Fig. S4** Principal component analysis (PCA) of leaf element concentrations in *Arabidopsis halleri*.
**Fig. S5** Multivariate analysis of the relationship between leaf and soil composition of *Arabidopsis halleri* individuals at their natural sites of growth.
**Fig. S6** Reproducibility of leaf Zn and Cd accumulation under standardized controlled growth chamber conditions in two independent experiments.
**Table S1** Redundancy models of leaf element concentrations on soil composition (total, extractable and exchangeable fractions)
**Table S2 **Linear regression models shown in Fig. 2
**Table S3** Redundancy models shown in Fig. S5
**Table S4** Composition of Zn‐ and Cd‐amended soil mix for plant cultivation under controlled growth conditions
**Methods S1** Detailed methods.
**Notes S4** Relationships between leaf concentrations of different minerals and between soil and leaf mineral composition.

**Notes S1** List of sampled populations.
**Notes S2** Leaf and soil data for each sampled plant individual.
**Notes S3** Results from principal component analyses (PCA) to test for a possible influence of sampling date on leaf composition.
**Notes S5** Leaf Zn and Cd concentrations of plant individuals cultivated in a Zn‐ and Cd‐amended soil mix under controlled conditions.
