# Supplementary material for: Relationships between soil and leaf mineral composition are element‐specific, environment‐dependent and geographically structured in the emerging model Arabidopsis halleri
Source: New Phytol. 2016 Oct 13;213(3):1274–86. doi: 10.1111/nph.14219 (PMC5248639; doi:10.1111/nph.14219)
Supplement: Supplementary file 1 — Fig. S1 Map of European sampling sites and edaphic range of Arabidopsis halleri. Fig. S2 Classification of sampling sites into metalliferous and nonmetalliferous according to soil composition. Fig. S3 Comparison of leaf element concentrations between Arabidopsis halleri populations at nonmetalliferous and metalliferous sites. Fig. S4 Principal component analysis (PCA) of leaf element concentrations in Arabidopsis halleri. Fig. S5 Multivariate analysis of the relationship between leaf and soil composition of Arabidopsis halleri individuals at their natural sites of growth. Fig. S6 Reproducibility of leaf Zn and Cd accumulation under standardized controlled growth chamber conditions in two independent experiments. Table S1 Redundancy models of leaf element concentrations on soil composition (total, extractable and exchangeable fractions) Table S2 Linear regression models shown in Fig. 2 Table S3 Redundancy models shown in Fig. S5 Table S4 Composition of Zn‐ and Cd‐amended soil mix for plant cultivation under controlled growth conditions Methods S1 Detailed methods. Notes S4 Relationships between leaf concentrations of different minerals and between soil and leaf mineral composition. [file NPH-213-1274-s001.pdf]

***New Phytologist* Supporting Information**

Article title: Relationships between soil and leaf mineral composition are element-specific, environment-dependent and geographically structured in the emerging model *Arabidopsis halleri*

Authors: Ricardo J. Stein, Stephan Höreth, J. Romário F. de Melo, Lara Syllwasschy, Gwonjin Lee, Mário L. Garbin, Stephan Clemens, Ute Krämer

Article acceptance date: 15 August 2016

The following Supporting Information is available for this article:

Methods S1 Detailed Methods.

Notes S4 Relationships between leaf concentrations of different minerals and between soil and leaf mineral composition.

Fig. S1 Map of European sampling sites and edaphic range of *A. halleri*.

Fig. S2 Classification of sampling sites into metalliferous and non-metalliferous according to soil composition.

Fig. S3 Comparisons of leaf element concentrations between *A. halleri* populations at non-metalliferous and metalliferous sites.

Fig. S4 Principal Component Analysis (PCA) of leaf element concentrations in *A. halleri.*

Fig. S5 Multivariate analysis of the relationship between leaf and soil composition of *A. halleri* individuals at their natural sites of growth.

Fig. S6 Reproducibility of leaf Zn and Cd accumulation under standardized controlled growth chamber conditions in two independent experiments.

Table S1 Redundancy models of leaf element concentrations on soil composition (total, extractable and exchangeable fractions).

Table S2 Linear regression models shown in Fig. 2.

Table S3 Redundancy models shown in Fig. S5.

Table S4 Composition of Zn- and Cd-amended soil mix for plant cultivation under controlled growth chamber conditions.

Methods S1. Detailed Methods. Plant and soil sampling in the field Locations of field sites hosting populations of *Arabidopsis halleri* (L.) O'Kane and Al-Shehbaz in Europe were assembled from the Global Biodiversity Information Facility (GBIF, http://www.gbif.org/), published records (Kolník & Marhold, 2006; Koch & Matschinger, 2007; Godé *et al.*, 2012; Pauwels *et al.*, 2012) and internet searches for historical or present Zn, Pb and Cu mines and smelters. At 165 field sites, we took one pair of a leaf and a soil sample from each sampled plant individual (Fig. S1, Notes S1; n = 3 to 20 individuals per site, averaging 12 individuals; minimum distance to sampled neighbour 3 m; 3 June to 29 October 2011, 30 July to 13 October 2012). Leaf samples (4 to 10 of the youngest undamaged fully expanded leaves) were washed thoroughly in deionized water. Soil samples (between 50 and 250 g) were taken using a stainless steel soil coring device (diameter 0.014 m) at 0.05 to 0.15 m depth within a 0.05 m radius around each plant individual. Samples were placed into paper bags and left to dry in ambient air.

Processing and analysis of samples After additional drying (60°C for ≥ 3 d, ambient air for ≥ 1 d) and homogenization (≤ 1 mm particle size by manually squeezing paper bags), a subsample of 10 to 25 mg leaf tissue was weighed into PTFE MPV-100 microwave vessels (MLS GmbH, Leutkirch, Germany), manually mixed with 3 ml 65% (w/w) HNO_3 (AnalaR, Merck Ltd,

Darmstadt, Germany), microwave-digested with temperature ramping to 180°C over 20 min and holding for 10 min (StarT-1500, MLS GmbH, Leutkirch, Germany), transferred into 15-ml round-bottom polypropylene screw-cap tubes (Sarstedt AG & Co, Nümbrecht, Germany) and filled up to a total of 10 ml with ultrapure water (Milli-Q, Merck Millipore, Darmstadt, Germany). After microwave digestion of leaf tissues, all samples were fully in solution. Air-dried soil samples were sieved (2 mm mesh size). Protocols used for soil extractions were slightly modified from published protocols in order to accommodate high sample numbers and the broad variety of elements to be analysed: The total fraction (nominal) was extracted by mixing a 0.25-g subsample of soil in a mix of 2.25 ml of 37% (w/w) HCl (AnalaR, Merck Ltd, Darmstadt, Germany) and 0.75 ml of 65% (w/w) HNO_3, microwave digestion with temperature ramping to 160°C over 15 min and holding for 15 min (Chen & Ma, 2001). Extracts were filtered through filter paper (Whatman No. 1, Brandt, Wertheim, Germany) and adjusted to a total volume of 10 ml with ultrapure water. For the determination of extractable concentrations of elements, 1 g of soil was mixed with 10 ml of 0.1 M HCl in 15-ml round-bottom polypropylene screw-cap tubes using an overhead shaker (150 rpm at RT) for 1 h (Giancoli Barreto *et al.*, 2004; Menzies *et al.*, 2007; Deinlein *et al.*, 2012; Hanikenne *et al.*, 2013). For the measurement of exchangeable concentrations of elements, subsamples of 1 g soil was mixed with 10 ml of 0.01 M $BaCl_2$ in 15-ml round-bottom polypropylene screw-cap tubes using an overhead shaker (150 rpm at RT) overnight (Hendershot & Duquette, 1986; Menzies *et al.*, 2007). These soil extracts were filtered through Whatman No. 1 filter paper, followed by the addition of 1 ml 65% (w/w) HNO_3. Element concentrations (Al, B, Ca, Cd, Cr, Cu, Fe, K, Mg, Mn, Ni, P, Pb, S and Zn) were determined in technical triplicates by Inductively Coupled Plasma Atomic Emission Spectrometry (ICP-AES; iCAP 6500 Duo; ThermoFisher, Dreieich, Germany). Every 40 to 50

samples, we measured a blank and quality controls (QCs) of an intermediate multi-element calibration standard solution, the appropriate certified reference leaf or soil material (Polish Virginia Tobacco Leaves, INCT-PVTL-6, Institute of Nuclear Chemistry and Technology, Warsaw, Poland; San Joaquin Soil - 2709a and Hard Rock Mine Waste – 2780, National Institute of Standards and Technology, USA), and either a bulk homogenate of *A. halleri* leaf tissue, or both a metalliferous and a non-metalliferous soil, as internal laboratory-standardized reference materials (relative standard deviation RSD $\leq$ 5% among triplicate measurements, among means of independent measurements within a run, and among independent runs, in QCs for all elements). Among measurements of four independent subsamples of leaf material, relative standard deviation for elements present above trace levels was between 1 and 5%. Recoveries (San Joaquin Soil - 2709a) in extracts of the total (nominal; note that the true total fraction is not accessible with the method used) fractions were (%): Al (44), B (120), Ca (79), Cd (84), Cr (62), Cu (63), Fe (91), K (21), Mg (88), Mn (87), Ni (83), P (81), Pb (68), S (not given), Zn (89). Recoveries in leaf material (INCT-PVTL-6) were (%): Al (83), B (161), Ca (100), Cd (92), Cr (92), Cu (96), Fe (98), K (95), Mg (93), Mn (96), Ni (32), P (100), S (79), Pb (89), Zn (93). Note that the concentrations of some elements in certified reference materials were far lower than in most of the samples analysed in this study. **Data validation and analysis** To identify leaf samples potentially contaminated with trace amounts of soil, we generated a dataset of an artificially contaminated (0%, 0.001%, 0.01%, 0.1% and 1% w/w soil) leaf sample (n = 3 replicates) by spiking with extracts from each of 50 soil samples from this survey (25 metalliferous and 25 non-metalliferous soils chosen to reflect the diversity in soil composition) prepared using the leaf digestion method (see above). A Principal Component Analysis (PCA) was conducted on standardized (z-scores) $\mathrm{Log}_{10}(x + 1)$ element concentrations determined by

ICP-AES to identify elements that contributed the most to principal components, namely Al, Cr, Fe, Ni and Pb, using the function *rda* from the vegan (V. 2.0-10) R package. Logistic regression models with all five elements, their interactions and all possible element combinations were used to identify leaf samples containing traces of soil using the *lrm* and *validate.lrm* functions from the Design (V. 2.3-0) R package (Baxter *et al.*, 2008). To identify samples contaminated with trace amounts of soil, the initial dataset (2,006 leaf samples) was queried, and a total of 34 leaf samples were classified as soil-contaminated and excluded from further analysis. In order to classify sites and individual soil samples as metalliferous or non-metalliferous, element concentrations of individual soil samples or collection sites (median of soil samples) were $Log_{10}(x + 1)$-transformed, and PCAs were then conducted to identify the elements that consistently showed the largest differentiation in the first dimension irrespective of the soil fraction analysed: Cd, Cu, Pb and Zn (Fig. S2). Their standardized $Log_{10}(x + 1)$ concentrations (z-scores) were used to generate an Euclidean distance matrix using the function *vegdist* from the vegan (V. 2.0-10) R package, and clustering was performed using Ward's minimum variance with function *hclust* from the vegan (V. 2.0-10) R package. This procedure was performed on the total, extractable and exchangeable Cd, Cu, Pb and Zn concentrations, and cophenetic correlations and average silhouette widths were calculated (functions *cophenetic,* using the stats V. 2.15.3 R package, and *silhouette* from the cluster V. 2.0.1 R package) to decide which fraction (total, exchangeable and extractable soil element concentrations) allowed the most robust assignment of sites to either metalliferous or non-metalliferous character (Fig. S2a, c, e). Accordingly, we obtained 46 metalliferous and 119 non-metalliferous sites based on soil extractable concentrations of Cd, Cu, Pb and Zn (Fig. S2b, d, f; Notes S1), as well as 506 metalliferous and 1,466 non-metalliferous soil samples (Notes S2). To examine whether the

timing of sampling may have influenced leaf composition, PCAs were conducted of plant composition (population median concentrations of all analysed elements in leaves were $Log_{10}(x + 1)$-transformed and standardized (*z*-scores)) and sampling date (day of year), separately for metalliferous sites and non-metalliferous sites, and jointly for all sites. For none of the principal components (PC) that were statistically significant according to the Kaiser-Guttman criterion did Pearson correlations or scores (loadings) suggest any relationships between sampling date and the concentration of any element in leaves (R^2 or scores ≤ 0.4, Notes S3). Linear regression analyses identified a positive correlation only between Log_{10}(leaf Ca concentrations) and sampling day of the year (all populations, $R^2 = 0.019$, $P < 0.05$, $n = 165$). Histograms displaying probability densities of leaf element concentrations from various datasets were generated using the function *multhist* (with the plotrix V. 3.5-12 R package). To identify univariate relationships between element concentrations in leaves of individuals and the adjacent soil, linear regressions were generated separately for individuals on metalliferous and non-metalliferous soils using $Log_{10}(x + 1)$-transformed element concentrations employing the function *lm* (stats V. 2.15.3 R package). For multivariate relationships between leaf and soil element concentrations, and soil pH, we performed Redundancy Analysis (RDA) models using the function *rda* from the vegan (V. 2.0-10) R package. Standardized (*z*-score) $Log_{10}(x + 1)$ soil concentrations (exchangeable, extractable, and total fractions, respectively) and soil pH were used as explanatory variables in models employing $Log_{10}(x + 1)$ leaf concentrations as response variables. The explanatory variables were selected using the *step* function of the stats (V. 2.15.3) R package, in both forward and backward directions, with significant models with the lowest AIC values being selected and used for final models. We computed adjusted R^2 using the function *RsquareAdj* from the vegan (V. 2.0-10) R package for the three different soil fractions (Table S1)(Peres-Neto *et al.*, 2006).

To assess the residual within-site variation, we extracted the residuals (using the function *residuals* from the stats V. 2.15.3 R package) from the RDA between the standardized $Log_{10}(x + 1)$ soil exchangeable element concentrations, standardized soil pH (both used as explanatory variables, selected as described above), and the leaf element concentrations (as response variables). The residuals were extracted, and 165 PCAs (using the function *rda* from the vegan V. 2.0-10 R package) were performed on a *per site* basis. The first three principal components were selected (in sum explaining between 71 and 89% of the total variation) from each of the 165 PCAs, and scores were extracted for all sites per element. **Zn and Cd accumulation phenotyping in the growth chamber** Seven or more months after transfer to growth facilities, cuttings were made of healthy mother plants by excising small rosettes (5 to 7 leaves), dipping into rooting powder (1% (w/w) indole-3-butyric acid; Rhizopon AA, Rhizopon, Alphen aan den Rijn, NL) and introducing into small round pots (Ø 50 mm x 35 mm) with a mix of 2:1 volumes turf:sand for 15 d, followed by transplantation into the experimental soil mix. To prepare the experimental soil mix, sand and a low-organic loamy soil (H. Lauterbach GmbH & Co. KG, Schwabach, DE) were dried at 60°C for 3 d, sieved to 5 mm mesh size, and mixed in a cement mixer to 33% (w/w) sand. Metals (Sigma-Aldrich, Merck Ltd., Darmstadt, DE) were added as ZnS (300 mg Zn kg^{-1}) and $CdCl_2$ x H_2O (5 mg Cd kg^{-1}) in ultrapure water, with 500 mL suspension/solution added per 1.5 kg soil, followed by mixing overhead in rotating 2-L screw-top plastic bottles on a Heidolph Reax 20/8 (Heidolph Instruments GmbH & Co. KG, Schwabach, DE) at 10 rpm and room temperature for 2 h. The mix was dried at 65°C for 16 to 24 h, followed by homogenization in a cement mixer for 0.5 to 1 h and sieving to 2 mm mesh size. Per genotype, each of five replicate clones was grown individually in a square plastic pot (60x60x80 mm) in 270 to 300 g of experimental soil mix in a climate-controlled growth chamber

(GroBank, Arabidopsis BB-XXL.3, CLF Plant Climatics GmbH, Wertingen, DE), at a light intensity of 90 µmol m^{-2} s^{-1}, 8-h days at 20°C; 18°C during nights, 60% rH. After six weeks on experimental soils (transparent plastic lid covers for initial 2 w), entire shoots were harvested, washed in ultrapure water, dried at 60°C (see above), homogenized in a Precellys 24 homogenizer using the 2-mL hard tissue homogenizing kit CK28 at 5,000 rpm, two times 30 s (Bertin Technologies, Saint Quentin en Yvelines Cedex, FR) and subsamples digested for multi-element analysis (see above). The experiment was repeated independently using a subset of genotypes, with the following differences: Rooting was conducted without rooting powder and hydroponically in 0.1x Hoagland solution (Weber *et al.*, 2004), cultivation was at 45 to 55 µmol m^{-2} s^{-1} (16 h days) in 70x70x70 mm pots (300 to 340 g experimental soil mix), and sample homogenization was done as described for field-collected leaves. **References Methods S1**

Baxter IR, Vitek O, Lahner B, Muthukumar B, Borghi M, Morrissey J, Guerinot ML, Salt DE. 2008. The leaf ionome as a multivariable system to detect a plant's physiological status. *Proceedings of the National Academy of Sciences of the United States of America* **105**(33): 12081-12086. **Chen M, Ma LQ. 2001.** Comparison of three *aqua regia* digestion methods for twenty florida soils. *Soil Science. Society of America Journal* **65**: 491-499. **Deinlein U, Weber M, Schmidt H, Rensch S, Trampczynska A, Hansen TH, Husted S, Schjoerring JK, Talke IN, Kramer U, et al. 2012.** Elevated nicotianamine levels in *Arabidopsis halleri* roots play a key role in zinc hyperaccumulation. *Plant Cell* **24**(2): 708-723. **Giancoli Barreto SR, Nozaki J, De Oliveira E, Do Nascimento Filho VF, Aragao PH, Scarminio IS, Barreto WJ. 2004.** Comparison of metal analysis in sediments using EDXRF and ICP-OES with the HCl and Tessie extraction methods. *Talanta* **64**(2): 345-354. **Godé C, Decombeix I, Kostecka A, Wasowicz P, Pauwels M, Courseaux A, Saumitou-Laprade P. 2012.** Nuclear microsatellite loci for *Arabidopsis halleri* (Brassicaceae), a

model species to study plant adaptation to heavy metals. *American Journal of Botany* **99**(2): e49-52. **Hanikenne M, Kroymann J, Trampczynska A, Bernal M, Motte P, Clemens S, Krämer U. 2013.** Hard selective sweep and ectopic gene conversion in a gene cluster affording environmental adaptation. *PLoS Genetics* **9**(8): e1003707. **Hendershot WH, Duquette M. 1986.** A simple barium chloride method for determining cation exchange capacity and exchangeable cations. *Soil Science Society of America Journal* **50**: 605-608. **Koch MA, Matschinger M. 2007.** Evolution and genetic differentiation among relatives of *Arabidopsis thaliana*. *Proceedings of the National Academy of Sciences of the United States of America* **104**(15): 6272-6277. **Kolník M, Marhold K. 2006.** Distribution, chromosome numbers and nomenclature conspect of *Arabidopsis halleri* (Brassicaceae) in the Carpathians. *Biologia (Bratislava)* **61**: 41-50. **Menzies NW, Donn MJ, Kopittke PM. 2007.** Evaluation of extractants for estimation of the phytoavailable trace metals in soils. *Environmental Pollution* **145**(1): 121-130. **Pauwels M, Vekemans X, Gode C, Frerot H, Castric V, Saumitou-Laprade P. 2012.** Nuclear and chloroplast DNA phylogeography reveals vicariance among European populations of the model species for the study of metal tolerance, *Arabidopsis halleri* (Brassicaceae). *New Phytologist* **193**(4): 916-928. **Peres-Neto PR, Legendre P, Dray S, Borcard D. 2006.** Variation partitioning of species data matrices: estimation and comparison of fractions. *Ecology* **87**(10): 2614-2625. **Weber M, Harada E, Vess C, Roepenack-Lahaye EV, Clemens S. 2004.** Comparative microarray analysis of *Arabidopsis thaliana* and *Arabidopsis halleri* roots identifies nicotianamine synthase, a ZIP transporter and other genes as potential metal hyperaccumulation factors. *Plant Journal* **37**: 269-281.

Supporting Notes S4. Relationships between leaf concentrations of different minerals and between soil and leaf mineral composition. A Principal Component Analysis (PCA) of leaf mineral composition identified three groups of co-varying elements. The first group consisted of the nutrients P, K, S, Mg, Ca and B, the second group comprised Fe, Al and Cr, and a third group included Cd and Pb (Fig. S4). Furthermore, distinct positions of datapoints from samples taken on metalliferous and non-metalliferous soils suggested a strong influence of local soil type, and consequently inter-element relationships were analysed in the context of soil mineral composition. A more comprehensive assessment of the complex multi-factorial relationships between soil and leaf composition by Redundancy Analysis (RDA) confirmed a strong overall influence of soil exchangeable concentrations particularly of Pb, and also of Cd, Cu, Mn and Zn on their respective concentrations in leaves of *A. halleri* (Fig. S5a, Table S3, see Fig. 2). We additionally found this for Ni, which can be present as a co-contaminant in the metalliferous soils of our study. With increasing soil pH, leaf Mn concentrations decreased and leaf Ca concentrations increased, thus following soil exchangeable concentrations of these nutrients, as was observed in other species (Marschner, 1995; Kochian *et al.*, 2005). Leaf Al concentrations did not increase with lower soil pH (Kochian *et al.*, 2005), suggesting that *A. halleri* might effectively restrict the accumulation of Al when it is highly bioavailable. The outcome of this global analysis was dominated by the enormous differences between metalliferous and non-metalliferous soils. Among metalliferous soils analysed separately, leaf Pb concentrations were positively related to soil exchangeable Pb concentrations, as expected (Fig. S5b; see Fig. 2c). In addition, leaf Pb and Zn concentrations were inversely related to soil exchangeable Ca concentrations. This suggests a possible competition of Ca, or a possible interference of high soil

pH, with leaf accumulation of Zn and Pb, but not of Cd. The dampening of the increase in leaf Zn concentrations with increasing soil Zn concentrations on metalliferous soils (see Fig. 2a) remained unexplained by RDA given the lack of any relationship between exchangeable soil Zn and soil Ca concentrations. Leaf Cd concentrations were correlated with sulphur concentrations in the exchangeable soil fraction. This may reflect a biochemical dependence on the abundance of this macronutrient for the extreme levels leaf Cd hyperaccumulation found on some metalliferous soils. To date, laboratory-based molecular mechanistic studies have not confirmed a biochemical dependence of Zn, Cd or Ni-related extreme traits on sulphur-containing metabolites, in particular phytochelatins (Schat & Kalff, 1992; Krämer *et al.*, 1996; Schat *et al.*, 2002; Meyer *et al.*, 2011), but none of these have addressed differences between accessions from moderately and highly metalliferous soils. Alternatively, soil S availability may positively influence Cd availability for *A. halleri*. However, this possibility is only hypothetical, because the measured exchangeable soil Cd and S concentrations are largely unrelated with one another, and similarly unrelated are leaf Cd concentrations with exchangeable soil Cd concentrations. Finally, heightened soil pH was associated not only with lower leaf Mn and Ni concentrations, as was also observed across all soils, but additionally with lowered leaf Cu accumulation. Overall, soil macronutrients, in particular S- versus Mg/P-richness, appeared to be the most important soil constituents influencing leaf composition on metalliferous soils, in conjunction with soil pH. Among plants on metalliferous soils, we detected an inverse relationship between leaf Ni and Ca, and a few weak positive relationships, most importantly between the concentration of S and Cd, between Pb, Cu and Zn, and between Ca and Fe in leaves. Leaf composition of *A. halleri* on non-metalliferous soils showed a dependence on soil pH, and – to a lesser degree – on the concentrations of a group of soil minerals (Mg, Ni, Cr, inversely related with Ca; Fig. S5c).

Heightened soil pH had a moderately negative effect on leaf Zn accumulation, whereas leaf Cd and Pb concentrations did not follow this trend. Taken together, a substantial proportion of the large within-species variation observed on non-metalliferous soils, in particular the variation in leaf Cd concentrations, remained unexplained by soil composition (see Fig. 2; Table S3). In plants from non-metalliferous soils, there was an inverse relationship between leaf Mn and Ca concentrations, and a weak positive relationship between the concentrations of Pb and Cd, which were positively related with soil Mg and inversely related with soil and leaf K levels. The inter-element relationships detected in leaf composition by this approach reflect a combination of the global physiological properties of *A. halleri* and contrasting composition of different soils hosting *A. halleri*. As such, they are informative about the outcome of ecological plant-soil interactions, but direct information on physiological properties of *A. halleri* can only be inferred from the cultivation of multiple genotypes under standardized growth conditions. **References Supporting Notes S4**

Kochian L, Pineros MA, Hoekenga OA. 2005. The physiology, genetics and molecular biology of plant aluminium resistance and toxicity. *Plant and Soil* **274**: 175-195. **Krämer U, Cotter-Howells JD, Charnock JM, Baker AJM, Smith JAC. 1996.** Free histidine as a metal chelator in plants that accumulate nickel. *Nature* **379**: 635-638. **Marschner H. 1995.** *Mineral Nutrition of Higher Plants, 2nd edn*. London: Academic Press Ltd. **Meyer CL, Peisker D, Courbot M, Craciun AR, Cazale AC, Desgain D, Schat H, Clemens S, Verbruggen N. 2011.** Isolation and characterization of *Arabidopsis halleri* and *Thlaspi caerulescens* phytochelatin synthases. *Planta* **234**(1): 83-95. **Schat H, Kalff MM. 1992.** Are phytochelatins involved in differential metal tolerance or do they merely reflect metal-imposed strain? *Plant Physiology* **99**(4): 1475-1480. **Schat H, Llugany M, Vooijs R, Hartley-Whitaker J, Bleeker PM. 2002.** The role of phytochelatins in constitutive and adaptive heavy metal tolerances in hyperaccumulator

and non-hyperaccumulator metallophytes. *Journal of Experimental Botany* **53**(379): 2381-2392.

Notes S1 List of sampled populations.

Notes S2 Leaf and soil data for each sampled plant individual.

Notes S3 Results from Principal Component Analyses (PCA) to test for an influence of sampling date on leaf composition.

Notes S5 Leaf Zn and Cd concentrations of plant individuals cultivated in a Zn- and Cd-amended soil mix under controlled conditions.

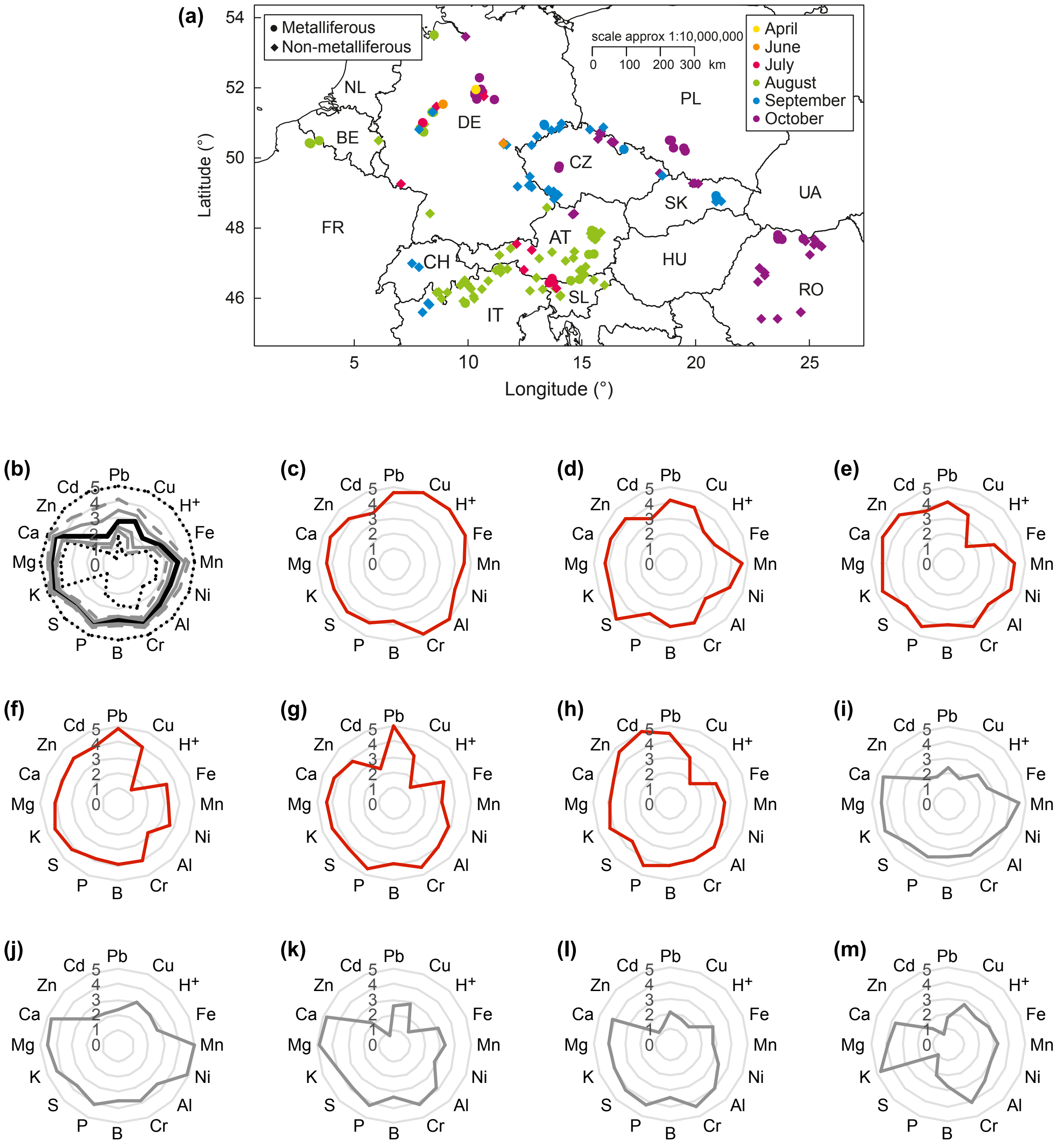

Fig. S1 Map of European sampling sites and edaphic range of *A. halleri.* (a) Symbols mark geographic positions of sampling sites on metalliferous (circles; *n* = 46) and non-metalliferous soils (diamonds; *n* = 119; see Fig. S2) and are colored by the month of sampling. (b) Edaphic range of *A. halleri* across Europe, shown as Log_{10} of scaled proton and exchangeable mineral concentrations in soils, with median (solid black), 25/75%iles (solid grey), 10/90%iles (dashed grey) and minima/maxima (dotted black) of 165 sites. (c-m) Edaphic diversity of sites hosting natural populations of *A. halleri* illustrated by examples of metalliferous sites (red) Băile Borșa/RO (c), Mina Suior/RO (d), Wulmeringhausen/DE (e), Ponte Nossa/IT (f), Haufenreith/AU (g), Miasteczko Śląskie/PL (h), and non-metalliferous sites (grey) Paisco Loveno/IT (i), Wallenfels/DE (j), Mutters/AU (k), Ukanc/SL (l), Lacul Balea/RO (m). Panels (c) to (m) show Log_{10} of scaled site medians of proton and exchangeable mineral concentrations in soils (d-i, l: *n* = 12; j: *n* = 11; c, k: *n* = 6, m: *n* = 3). Data were scaled through division by the maximum of all populations and multiplication with 10^5 (Maxima of all population medians; soil exchangeable concentrations [mg kg^{-1} DW]: Al 176, B 3.63, Ca 3,540, Cd 27.8, Cr 0.192, Cu 44.4, Fe 936, K 197, Mg 550, Mn 100, Ni 2.16, P 15.2, Pb 40.0, S 198, Zn 875; [mM] H^+ 1.66.

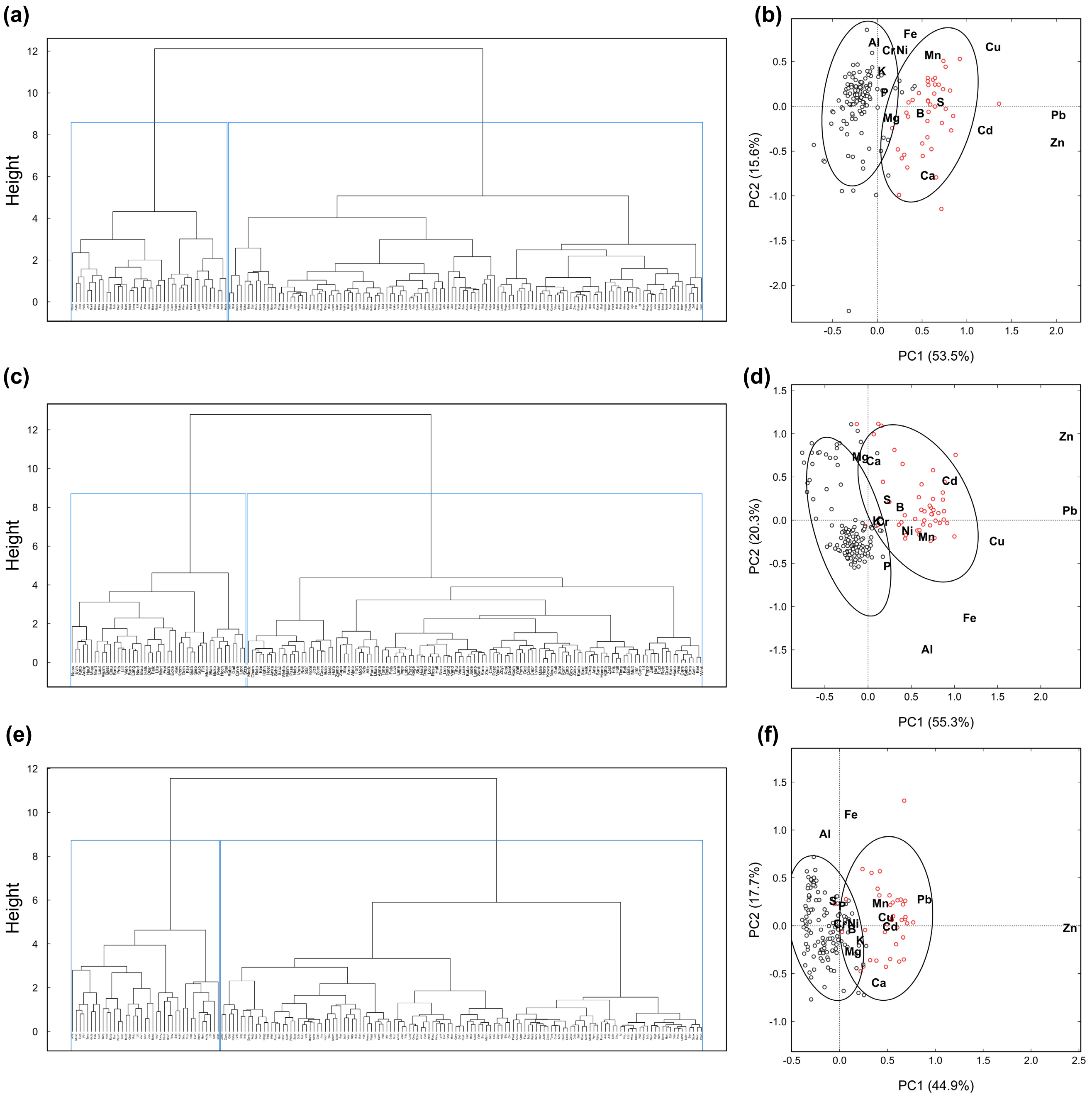

Fig. S2 Classification of sampling sites into metalliferous and non-metalliferous according to soil composition. (a, c, e) Ward clustering of soil Zn, Cd, Pb and Cu concentrations, and (b, d, f) the supporting Principal Component Analyses of concentrations of the full set of elements in the soil (Ca, K, Mg, P, S, B, Cu, Fe, Mn, Ni, Zn, Al, Cd, Cr and Pb), conducted on site median of $\mathrm{Log}_{10}(x + 1)$ soil concentrations in (a, b) total, (c, d) extractable and (e, f) exchangeable elements (n = 12 on average) for 165 sites. Sites grouped in cluster **I** (metalliferous)/cluster **II** (non-metalliferous) in (a), (c), and (e) (cophenetic correlation coefficients /average silhouette width: 0.81/0.63 (a), 0.86/0.75 (c), and 0.82/0.65 (e)), are represented by red/black symbols in (b), (d) and (f), respectively (elliptic lines at 95% confidence limits around group centroids).

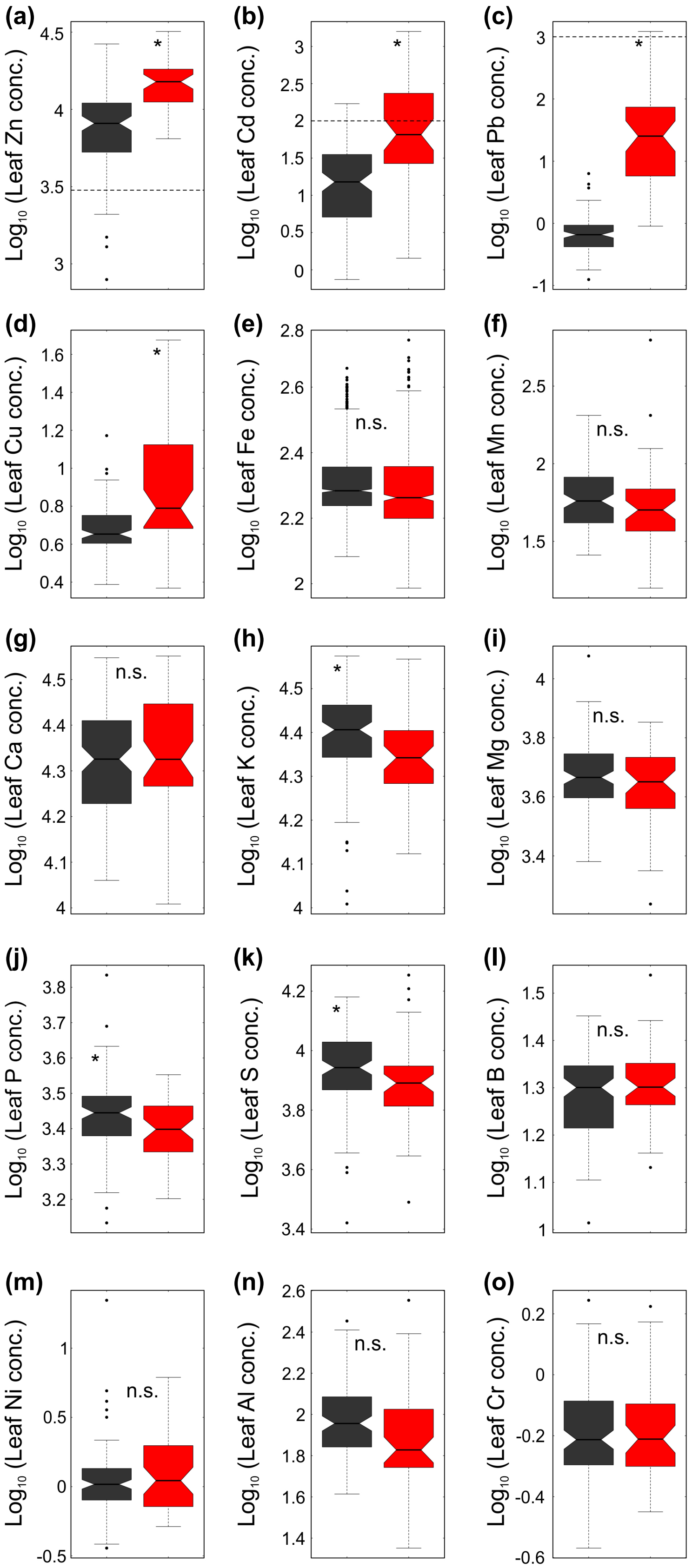

Fig. S3 Comparisons of leaf element concentrations between *A. halleri* populations at non-metalliferous and metalliferous sites. (a-o) Boxplots were generated from population medians of Log$_{10}$ leaf concentration of Zn, Cd, Pb, Cu, Fe, Mn, Ca, K, Mg, P, S, B, Ni, Al and Cr for non-metalliferous (black; n = 119) and metalliferous (red; n = 46) sites. Shown is the overall median (central horizontal line) with 1st and 3rd quartiles (box), 10th and 90th percentiles (horizontal bars), and outliers (filled circles; > 1.5-fold the interquartile range above/below the upper/lower quartile).
*: significant difference at $P < 0.05$ (general least squares); n.s.: not significant.

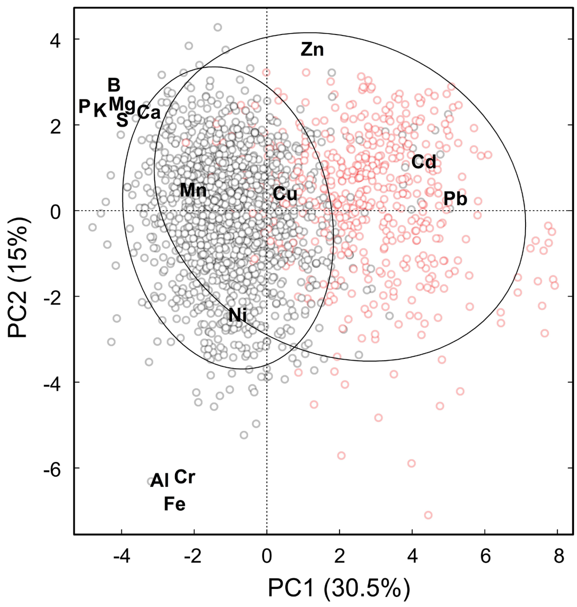

Fig. S4 Principal Component Analysis (PCA) of leaf element concentrations in *A. halleri*. Shown are datapoints for 1,972 individuals collected on non-metalliferous soils (n = 1,466 individuals; black) and metalliferous soils (n = 506 individuals; red) (elliptic lines at 95% confidence limits around group centroids). Leaf element compositions constituting PC1 and PC2 loadings are given in bold inside diagram. PCA was conducted employing standardized $Log_{10}(x + 1)$ leaf element concentrations (z-scores).

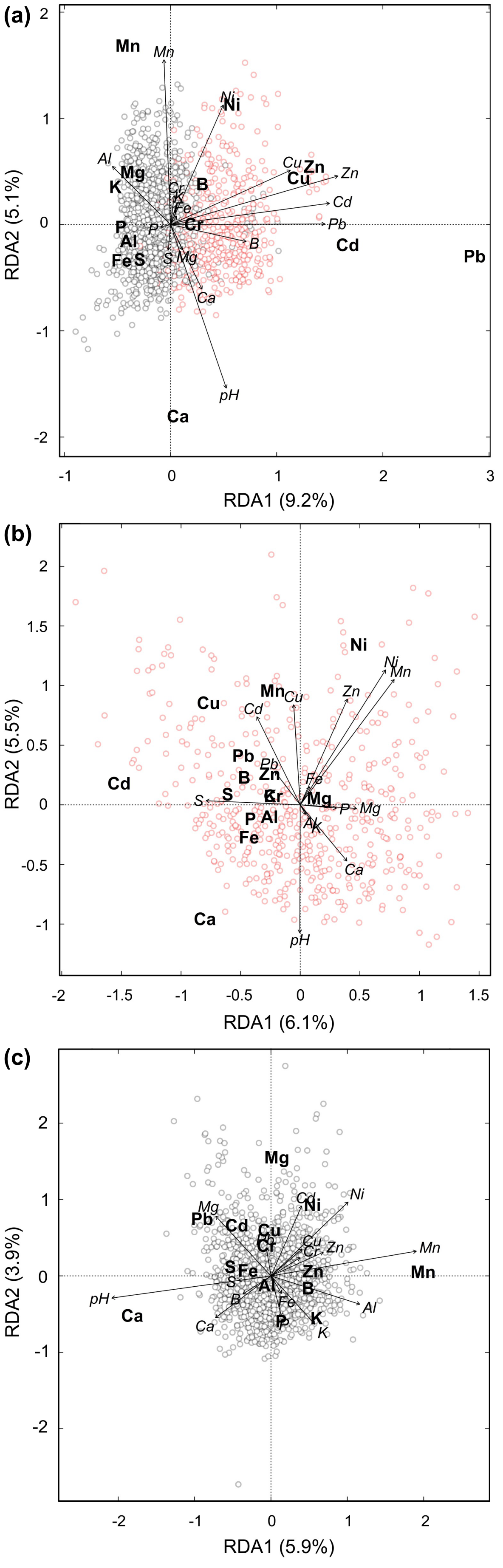

Fig. S5 Multivariate analysis of the relationship between leaf and soil composition of *A. halleri* individuals at their natural sites of growth. Diagrams show the first two dimensions of Redundancy Analysis (RDA) triplots for (a) the complete dataset (n = 1,972), and subsets of samples from (b) metalliferous soils (n = 506; red) and (c) non-metalliferous soils (n = 1,466; black). Shown are samples (open circles), leaf element concentrations (positions of bold characters), and their matrix correlations with soil exchangeable element concentrations/pH (arrows; characters in italics), for models based on standardized (z-scores) of $\text{Log}_{10}(x + 1)$ leaf element concentrations, of soil pH and of $\text{Log}_{10}(x + 1)$ soil exchangeable element concentrations (see Table S3).

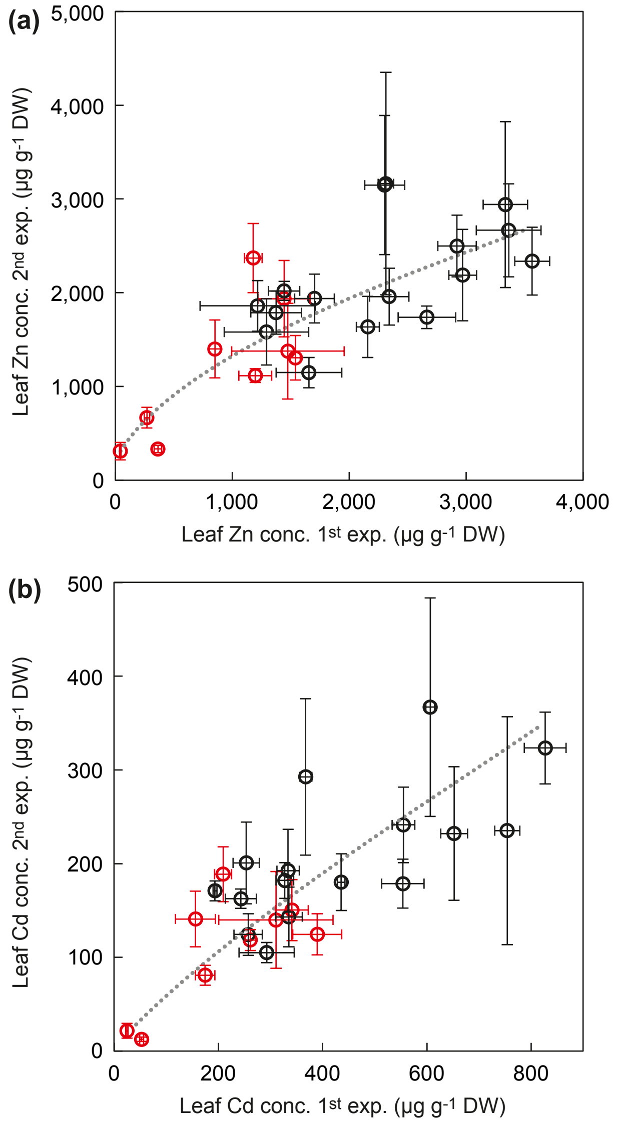

Fig. S6 Reproducibility of leaf Zn and Cd accumulation under standardized controlled growth chamber conditions in two independent experiments. Mean leaf (a) Zn and (b) Cd (± SD, n = 5) concentrations in a second independent experiment conducted in Bayreuth are plotted against mean leaf concentrations (± SD, n = 5) of the first experiment conducted in Bochum and shown in Fig. 3 and 4d. Dotted lines correspond to best fits $y = 30.8\ x^{0.545}$ (R^2 = 0.76) (a) and $y = 1.26\ x^{0.838}$ (R^2 = 0.77) (b) of means for 25 genotypes (9 originating from metalliferous soils, shown in red; 16 originating from non-metalliferous soiles, black).

Table S1 Redundancy models of leaf element concentrations on soil composition (total, extractable and exchangeable fractions).

**Redundancy Analysis Model**	**Predictor variables selected**[§]	***n***	**Adjusted R^2**	***F*-value**	***P*-value**
rda (**Y** = Leaf element conc., **X** = Soil exchangeable element conc.)	Zn + Mn + Cd + Ni + Ca + Mg + Pb + P + S + K + Cu + Fe + Al + Cr + B	1,972	0.271	48.523	< 0.005
rda (**Y** = Leaf element conc., **X** = Soil extractable element conc.)	Cd + Cu + Mg + Zn + Ni + Ca + Al + S + P + Pb + Mn + B + K + Fe + Cr	1,972	0.236	40.806	< 0.005
rda (**Y** = Leaf element conc., **X** = Soil total element conc.)	Cd + Ca + Cu + Ni + Zn + Mg + P + Al + Pb + S + K + Mn + Fe + B + Cr	1,972	0.237	39.343	< 0.005

[§]listed in the order of decreasing explanatory power

Table S2 Linear regression models shown in Fig. 2.

**Linear Regression Model**	***df***	***a***	***b***	***RSE***[§]	R^2	***F*-value**	***P*-value**
[Non-metalliferous] Leaf Zn conc. = *a**(soil exchangeable Zn conc.) + *b*	1,464	0.272	3.834	0.295	0.320	689.700	< 0.005
[Metalliferous] Leaf Zn conc. = *a**(soil exchangeable Zn conc.) + *b*	504	0.028	4.122	0.193	0.007	4.437	0.0357
[Non-metalliferous] Leaf Cd conc. = *a**(soil exchangeable Cd conc.) + *b*	1,464	0.178	1.472	0.623	0.035	54.230	< 0.005
[Metalliferous] Leaf Cd conc. = *a**(soil exchangeable Cd conc.) + *b*	504	0.186	1.950	0.689	0.047	26.100	< 0.005
[Non-metalliferous] Leaf Pb conc. = *a**(soil exchangeable Pb conc.) + *b*	1,464	0.204	0.038	0.445	0.052	81.200	< 0.005
[Metalliferous] Leaf Pb conc. = *a**(soil exchangeable Pb conc.) + *b*	504	0.519	1.143	0.714	0.222	145.000	< 0.005
[Non-metalliferous] Leaf Cu conc. = *a**(soil exchangeable Cu conc.) + *b*	1,464	0.043	0.734	0.226	0.017	26.230	< 0.005
[Metalliferous] Leaf Cu conc. = *a**(soil exchangeable Cu conc.) + *b*	504	0.191	0.866	0.317	0.162	98.510	< 0.005
[Non-metalliferous] Leaf Fe conc. = *a**(soil exchangeable Fe conc.) + *b*	1,464	0.072	2.187	0.245	0.022	33.740	< 0.005
[Metalliferous] Leaf Fe conc. = *a**(soil exchangeable Fe conc.) + *b*	504	-0.001	2.210	0.306	-0.002	0.001	0.972
[Non-metalliferous] Leaf Mn conc. = *a**(soil exchangeable Mn conc.) + *b*	1,464	0.219	1.604	0.239	0.240	463.700	< 0.005
[Metalliferous] Leaf Mn conc. = *a**(soil exchangeable Mn conc.) + *b*	504	0.198	1.554	0.339	0.102	58.590	< 0.005

[§]Residual Standard Error

Note that for all elements, separate models are provided for metalliferous and non-metalliferous soil samples for ease of comparison.

Table S3 Redundancy models shown in Fig. S5.

Redundancy Analysis Model	Predictor variables selected§	*n*	Adjusted R^2	*F*-value	*P*-value
[Complete dataset] rda (**Y** = Leaf element conc., **X** = Soil exchangeable element conc. + soil pH)	Zn + pH + Cd + Ni + Pb + P + S + K + Mg + Ca + Cu + Al + Mn + Fe + Cr + B	1,972	0.236	38.954	< 0.005
[Non-metalliferous] rda (**Y** = Leaf element conc., **X** = Soil exchangeable element conc. + soil pH)	pH + Cd + Zn + Ni + Ca + Mg + P + Mn + Pb + K + S + Cr + Al + Fe + Cu + B	1,466	0.193	22.866	< 0.005
[Metalliferous] rda (**Y** = Leaf element conc., **X** = Soil exchangeable element conc. + soil pH)	Mn + Cd + S + K + pH + Ni + Pb + Mg + Cu + Al + Ca + Fe + Zn + P	506	0.246	11.305	< 0.005

§listed in the order of decreasing explanatory power

Table S4 Composition of Zn- and Cd-amended soil mix for plant cultivation under controlled growth chamber conditions.

Element	Total		Extractable		Exchangeable	
	mean	SD	mean	SD	mean	SD
**Cd**	5.34	0.39	4.10	0.33	0.027	0.003
**Zn**	318	28	13.9	3.2	0.239	0.083
**Al**	11,900	1,500	208	15	0.612	0.237
**B**	43.0	1.0	9.87	0.06	0.245	0.007
**Ca**	3,710	220	2,990	320	1,290	90
**Co**	6.53	1.49	0.669	0.056	n.d.	n.d.
**Cr**	19.9	3.7	0.254	0.035	0.006	0.001
**Cu**	4.38	2.52	1.69	0.18	n.d.	n.d.
**Fe**	11,600	1,700	118	9	0.606	0.169
**K**	2,540	270	215	13	164	16
**Mg**	2,250	130	353	46	98.0	6.4
**Mn**	395	89	122	15	6.95	1.54
**Mo**	0.94	0.31	0.068	0.002	0.029	0.001
**Ni**	14.0	2.5	0.868	0.037	n.d.	n.d.
**P**	488	46	115	9	3.40	0.26
**Pb**	14.3	1.1	3.38	0.16	0.01	0.00
**S**	336	23	27.1	2.5	25.6	2.1
**pH**	7.19	0.02				

Element concentrations given in mg kg^{-1} dry soil; *n* = 8 replicate, independently prepared soil mixes; n.d. not detectable.
